# Biomarkers in Breast Cancer: An Old Story with a New End

**DOI:** 10.3390/genes14071364

**Published:** 2023-06-28

**Authors:** Lyvia Neves Rebello Alves, Débora Dummer Meira, Luiza Poppe Merigueti, Matheus Correia Casotti, Diego do Prado Ventorim, Jucimara Ferreira Figueiredo Almeida, Valdemir Pereira de Sousa, Marllon Cindra Sant’Ana, Rahna Gonçalves Coutinho da Cruz, Luana Santos Louro, Gabriel Mendonça Santana, Thomas Erik Santos Louro, Rhana Evangelista Salazar, Danielle Ribeiro Campos da Silva, Aléxia Stefani Siqueira Zetum, Raquel Silva dos Reis Trabach, Flávia Imbroisi Valle Errera, Flávia de Paula, Eldamária de Vargas Wolfgramm dos Santos, Elizeu Fagundes de Carvalho, Iúri Drumond Louro

**Affiliations:** 1 Núcleo de Genética Humana e Molecular, Departamento de Ciências Biológicas, Universidade Federal do Espírito Santo (UFES), Vitória 29075-910, ES, Brazil; 2 Programa de Pós-Graduação em Biotecnologia, Universidade Federal do Espírito Santo, Vitória 29047-105, ES, Brazil; 3 Instituto Federal de Educação, Ciência e Tecnologia do Espírito Santo (Ifes), Cariacica 29150-410, ES, Brazil; 4 Centro de Ciências da Saúde, Curso de Medicina, Universidade Federal do Espírito Santo (UFES), Vitória 29090-040, ES, Brazil; 5 Escola Superior de Ciências da Santa Casa de Misericórdia de Vitória (EMESCAM), Vitória 29027-502, ES, Brazil; 6 Instituto de Biologia Roberto Alcântara Gomes (IBRAG), Universidade do Estado do Rio de Janeiro (UERJ), Rio de Janeiro 20551-030, RJ, Brazil

**Keywords:** breast cancer, cancer genetics, biomarkers, personalized medicine

## Abstract

Breast cancer is the second most frequent cancer in the world. It is a heterogeneous disease and the leading cause of cancer mortality in women. Advances in molecular technologies allowed for the identification of new and more specifics biomarkers for breast cancer diagnosis, prognosis, and risk prediction, enabling personalized treatments, improving therapy, and preventing overtreatment, undertreatment, and incorrect treatment. Several breast cancer biomarkers have been identified and, along with traditional biomarkers, they can assist physicians throughout treatment plan and increase therapy success. Despite the need of more data to improve specificity and determine the real clinical utility of some biomarkers, others are already established and can be used as a guide to make treatment decisions. In this review, we summarize the available traditional, novel, and potential biomarkers while also including gene expression profiles, breast cancer single-cell and polyploid giant cancer cells. We hope to help physicians understand tumor specific characteristics and support decision-making in patient-personalized clinical management, consequently improving treatment outcome.

## 1. Introduction

Breast cancer (BC) is the main cause of cancer death affecting women worldwide and the second most frequent cancer overall [1]. It is known to be a heterogeneous disease both clinically [2] and molecularly [3].

In the era of personalized medicine, traditional prognostic markers, such as lymph node metastasis, tumor size and histological tumor grade are no longer sufficient to guide early-diagnosed BC patients [4]. Recent technology advances improved our understanding of the molecular basis of tumor progression and treatment responses [5]. The identification of molecular biomarkers that may be useful as prognostic and predictive markers has helped clinicians in therapeutical decisions, conducting treatment with a more individualized approach and consequently optimizing therapy, as well as avoiding overtreatment, undertreatment, and incorrect treatment [2]. Prognostic markers can help clinicians predict tumor aggressiveness and invasiveness, allowing for better treatment decision [6,7].

This review summarizes traditional, novel, and potential prognostic biomarkers and gene expression profiles applied to breast cancer. In addition, we discuss limitations and future potentials biomarkers, such as single-cell and polyploid giant cancer cells (PGCCs), and how they can help clinicians understand this heterogeneous disease and decide on more personalized treatments.

## 2. Lymph Node Metastasis, Tumor Size, and Histological Tumor Grade

Lymph node metastasis (LNM), tumor size, and histological tumor grade are the best-established traditional prognostic factors in BC [8]. The detection of LNM affects disease management, staging, and treatment. Breast cancer LNM can be classified into the following categories: N0 (no cancer cells in nearby lymph nodes); N1 (cancer has spread to 1–3 underarm lymph nodes, or a few cells have been found in lymph nodes near the breastbone during sentinel node biopsy); N2 (cancer has spread to 4–9 underarm lymph nodes, or mammary lymph nodes are enlarged); and N3 (cancer has spread to 10 or more axillary lymph nodes, and one site is larger than 2 mm; or cancer is found in lymph nodes under the collarbone, and at least one site is larger than 2 mm). The absolute number of lymph nodes involved is also of prognostic importance: patients with four or more involved lymph nodes have a worse prognosis than those with fewer than four affected lymph nodes [9].

Historically, lymph node involvement was primarily verified by a surgical procedure, with secondary treatments based on pathological analysis. Although this sequence is still used in many cases, in the past two decades, new adjuvant treatments are increasingly being used as initial treatments [10]. Imaging tests before and after adjuvant treatments can guide treatment next steps, suggesting new drugs or surgery [11]. Ultrasound (US)-guided biopsies help define LNM extension. Adjuvant treatments can decrease tumor size and lymph node involvement, decreasing the need for surgery and turning an inoperable tumor into an operable one [10].

Axillary LNM is associated with local or distant metastatic recurrence [12], being an important factor in determining BC stage and deciding postoperative treatment [13]. To assess lymph node involvement, a sentinel lymph node biopsy (SLNB) is used as initial procedure. However, new research shows that SLNB is recommended if there is a limited nodal involvement [10]. In addition, SLNB was considered too invasive in patients with small primary BC because LNM is unlikely [14]. In extensive nodal involvement, either medical neoadjuvant systemic therapy (NST) or axillary lymph node dissection (ALND) are recommended over SLNB [10]. After NST, imaging tests are performed to monitor treatment response. In good responses (zero-two nodes), a target axillary dissection is recommended, and in poor responses (≥three nodes), ALND is the standard option [15]. In both cases, treatment based on tumor biology, and residual disease is necessary.

To provide the most personal treatment for BC with LNM, a multidisciplinary approach is required. A combination of local and systemic treatments, such as radiation therapy, chemotherapy, targeted therapy, surgery and endocrine therapy is used in a complementary way [10]. In the same way, NST allied with surgery is the recommended approach in lymph node involvement in order to improve patient survival and treatment response [10].

In most BC types, the presence of LNM can be predicted by tumor size [16]. Despite not being a direct correlation (the larger the tumor, the more lymph nodes involved) it is suggested as a biological relationship [16]. Furthermore, tumor size prognostic impact is worsened by lymph node involvement [12].

Accurate tumor sizing is determinant to guide treatment options. Imaging tools, such as US, mammography and magnetic resonance imaging (MRI), are used to determine tumor size. Cuesta et al. [17] comparatively analyzed these three imaging methods in order to verify which one is the most accurate for determining BC tumor size, determining that MRI is the best tool [17,18]. Moreover, the histological BC subtype can influence the size estimation in imaging exams, which must be considered when planning patient treatment [17].

The largest size obtained by imaging exams is an important factor in deciding which type of surgery is performed—mastectomy or breast conservation. Usually, MRI is recommended in high-risk patients, in invasive lobular BC, and in dense breast tissue [19,20]. Haraldsdottir et al. [21] showed that the US tends to underestimate invasive BC size in 10.3% of patients by 10 mm or more, therefore the US interpretation using mammography can lower underestimation risk [21]. In contrast, MRI may overestimate tumor extent [22]. Together, mammography and MRI are more sensitive than other tests or combination of tests [22].

Usually, mastectomy is the chosen surgical procedure for advanced-stage breast cancer [23]. Gu et al. [23] suggests that breast conservation surgery is performed in small primary breast cancer. Women who are concerned about BC recurrence often choose mastectomy [23]. Both procedures are safe and routinely used [24]. Information such as tumor size, lymph node involvement, cancer stage and personal beliefs should be considered when choosing the surgical procedure to be performed.

The histological tumor grade represents the morphological assessment of tumor biology [8]. According to Nottingham Grading System three histological grades in BC are known: well differentiated (grade I); moderately differentiated (grade II); and poorly differentiated/most aggressive (grade III) [25]. The Nottingham Grading System is based on three dimensions: degree of tubule or gland formation; nuclear pleomorphism; and mitotic count. Each dimension is scored from 1 to 3, and tumors with higher grades are associated with lower survival [26,27].

Usually, half of BC cases are grade I or III [27]. Grade III tumors are prescribed adjuvant chemotherapy, while grade I tumor are normally estrogen-receptor-positive [8]. Grade II tumors are very heterogeneous and related to intermediary recurrence risk [28]. Wang et al. [27] proposed a method that divides grade II tumors into low- and high-risk, which helps reduce under- and overtreatment [27]. In this way, genetic biomarkers studies suggest a reclassification of grade II tumors in two subgroups, one more similar to grade I and one more similar to grade III [29], which could help in clinical guidelines.

## 3. Molecular Predictive and Prognostic Markers

### 3.1. Classical Markers

#### 3.1.1. Ki-67

Ki-67 is a nuclear and nucleolar nonhistone protein and, in humans, is encoded by the *MKI-67* gene mapped to chromosome 10q26.2 [30]. Ki-67 expression is related to cell proliferation, and higher protein levels are related to biological aggressiveness in BC [30,31]. The prognostic value of Ki-67 staining can be a useful tool for predicting survival and recurrence rates and, when associated with other markers, can also be used for primary tumor classification and metastases [32]. In clinical practice, its use has attracted a lot of attention, especially in hormone receptor (HR)-positive cases, as a discriminator between luminal A and B types, with luminal B generally being more proliferative and having a higher Ki-67 detection than luminal A [31,33].

In neoadjuvant endocrine therapy (NET), Ki-67 measurement after a short treatment reveals a biological response to the therapy, which is the most used metric for evaluating results [31]. During the IMPACT trial, which aimed to compare the recurrence and risk of death of HR-positive BC patients on three different NET regimens [34], changing Ki-67 levels was used as one of the main endpoint biomarkers. In this study, after 2 and 12 weeks using anastrozole and tamoxifen, Ki-67 suppression was greater with anastrozole (76% and 82%) than with tamoxifen (60% and 62%) and the combination of anastrozole and tamoxifen (64% and 61%) [35]. Short-term Ki-67 changes can predict long-term benefits and outcomes, allowing for the response evaluation of specific therapies [31,34,35].

Owing to doubts about its analytical validity, Ki-67 is still not widely used in clinical routines [34]. This is due to a lack of consensus about scoring methods and cutoff values caused by the great variability of interlaboratory scoring approaches and reliability of different antibodies [31,36]. Currently, guidelines are needed for the use of Ki-67 in clinical practice in order to achieve scoring uniformity, standardization and subsequent clinical validation [34].

#### 3.1.2. ER

Estrogen receptor (ER) is a nuclear receptor that acts as a ligand-activated transcription factor [37]. Two isoforms of ER are present in the nucleus: ERα and ERβ [38]. In BC, the main form of ER is ERα, which functions as a transcription factor for genes associated with cell survival and proliferation [39]. The role of ERβ has not yet been fully understood, with divergent functions being related to this isoform [33]. In this review, ER is used in reference to ERα/*ESR1*.

ER is the most well-established common predictive marker used in BC, mainly for its classification and treatment option using endocrine therapy (ET) [31]. ER measurement is mandatory and recommended in newly diagnosed BC cases [3,40]. ER expression is recognized as a BC biomarker of favorable prognosis when compared to ER-negative cases [31,33]. Response to ET depends on ER positivity and varies according to ER tumor expression levels [31].

Estrogen suppression treatments use ER antagonists to kill ER-positive BC cells [41]. Several ETs are approved and routinely used for the adjuvant treatment of ER-positive BC patients and have demonstrated an improvement in survival and time to disease recurrence [38,41]. In luminal-type BC, which expresses both ER and progesterone receptor, after surgery, adjuvant ET is standard and recommended for at least 5 years [3].

Among different types of ET, aromatase inhibitors (AIs), such as anastrozole, letrozole, or exemestane, act by blocking estrogen biosynthesis, decreasing circulating estrogen levels in postmenopausal patients [38,40,41]. On the other hand, selective estrogen receptor modulators (SERMs), such as tamoxifen, are indicated for premenopausal patients act by competing with estrogen for ER binding and may have antagonistic activity in breast tissue [38,41]. Selective estrogen receptor degraders (SERDs), such as fulvestrant, have ER degrading and antagonistic effects and, in the breast, have anti-estrogenic effects [42].

Nonetheless, ET resistance can influence therapy results. The most common case of acquired resistance is due to estrogen-independent ER reactivation, due to specific *ESR1* gene mutations, rarely found in primary BC and more frequently found in recurrent and metastatic cases, especially after long-term AI treatment [38,40,41,42]. Because they do not act through the same mechanisms, acquired resistance to a specific drug can be circumvented by using other classes of ET, which can be used sequentially to treat ER-positive cases [40]. Currently, some ET treatments also target other molecules, such as CDK4/6, PI3K or mTORC1 [43].

#### 3.1.3. PR

The progesterone receptor (PR), like ER, is a member of the nuclear receptor family that functions as ligand-activated transcription factors [33]. When active, PR binds to DNA and regulates the expression of several cell cycle genes, cell differentiation, and proliferation [44]. ER-positive BC shows positivity for PR in approximately 80–90% of cases [31]. Generally, PR measurement is performed together with ER, being mandatory and recommended in newly diagnosed cases and in recurrent and metastatic lesions [40]. Like ER, immunohistochemistry (IHC) is the recommended assay for PR evaluation [31].

Currently, the benefit of measuring PR is not yet fully understood and remains controversial [44] because this receptor can be induced by estrogen [40]. This occurs because the *PGR* gene is regulated by ER as an ER-dependent gene product, causing crosstalk between these two receptors [31]. Thus, the presence of PR works as a biomarker that indicates a functional and intact ER pathway [40,44], which directly impacts a tumor’s ability to respond to endocrine therapies (ETs).

In addition to being related to a functioning ER pathway, PR positivity also shows a better response to ETs, and PR-positive tumor patients generally have better clinical outcomes [45]. High PR expression may be related to a better tamoxifen response, lower recurrence rate, and longer disease-free survival [44].

Semi-quantitative PR score, obtained by tests such as PAM50, helps discriminate BC types, such as luminal A and B [46]. Thus, high PR expression is observed more commonly in Luminal A subtypes, which show better prognosis than Luminal B [46].

Mohammed et al. [47] showed that PR, in the presence of an agonist ligand, can associate with ERα, modulating its expression and directing its binding to chromatin. This modulation of gene expression is associated with good prognosis [47]. Studies have shown that ER and PR can oppose or cooperate with each other, and understanding this crosstalk will aid in the development of better therapies [44].

Selective progesterone receptor modulators (SPRMs) are being studied in clinical trials to modulate and induce agonist, antagonist, or mixed PR responses in a tissue-specific manner [48]. Among these modulators are mifepristone, telapristone acetate and onapristone [49]. Mifepristone and onapristone are antiprogestogens that have had positive responses in patients who did not respond to other types of treatments [49]. Gaddy et al. [50] demonstrated that mifepristone, alone or in combination with 4-hydroxytamoxifen (4-OHT), promoted cell death and the growth arrest of ER/PR-positive MCF7 cells that were resistant to antiestrogens [50].

#### 3.1.4. HER2

HER2 (human epidermal growth factor receptor 2), encoded by the *ERBB2* gene, is a member of the human epidermal growth factor receptor family, along with HER1, HER3, and HER4 [3,51]. In BC, *ERBB2* amplification and consequent overexpression occurs in 13–15% of cases and is related to a worse prognosis due to the high metastatic potential of HER2-positive tumors [3,31,52]. HER2 status is determined by immunohistochemistry (IHC) and/or in situ hybridization (ISH) [31].

HER2 activation occurs through dimerization after ligand binding, although a specific ligand for HER2 is not known [3]. HER2 signaling leads to tumor growth and proliferation, adhesion, cell survival and metastasis, which are related to the activation of pathways such as RAS and PI3K/AKT/MAPK [3,40]. HER2 overexpression leads to histological characteristics of aggressiveness, being associated with a shorter survival time [31].

HER2 status measurement is mandatory in cases of invasive BC and recommended in cases of recurrence and metastasis [3,40]. For HER2 assessment, IHC reveals a response based on HER2 overexpression in a score ranging from 0 to 3+, where 0/1+ is considered negative, 3+ is considered positive, and 2+ is considered ambiguous, requiring additional evaluation using FISH [31]. Tumors with a score of 3+ exhibit increased cell proliferation and invasion activity [52].

Anti-HER-2 targeting therapies have shown efficacy in BC cases marked by *ERBB2* amplification or the overexpression of the HER protein [3]. These therapies currently involve the use of drugs based on anti-HER2 monoclonal antibodies, such as trastuzumab, pertuzumab, and margetuximab; tyrosine kinase inhibitors (TKIS) such as lapatinib, tucatinib and neratinib; and antibody-drug conjugates (ADCs), which bind a cytotoxic agent to a monoclonal antibody, such as trastuzumab deruxtecan (T-DXd) and ado-trastuzumab emtansine (T-DM1) [31,53]. Initial therapy for metastatic HER-2 tumors uses a combination of two HER2 antibodies: pertuzumab and trastuzumab, associated with a taxane [54].

Intra- and intertumoral HER2 heterogeneity seems to negatively affect the response to anti-HER2 therapy, leading to shorter recurrence time and patient survival, increased tumor size, worse histology, and greater number of lymph node metastases [53]. In addition, change in HER2 status after metastasis affects therapeutic strategies. In metastatic tumors, the loss of HER2 occurs more frequently [3]. In brain metastases cases, tumor resistance is due to the difficulty in penetrating the blood–brain barrier, and generally, these patients are excluded from anti-HER2 clinical trials [3,53]. Notably, TKIs have a smaller size and greater penetration capacity, making lapatinib, tucatinib, and neratinib a better option for patients with brain metastases [53].

#### 3.1.5. p53

p53 is a tumor suppressor protein, encoded by the *TP53* gene, which is involved in transcriptional regulation related to cell cycle arrest, differentiation, senescence, apoptosis, cell growth, and DNA repair [55,56]. As an important tumor suppressor, its degradation is directly linked to tumor formation, progression, and metastasis [56].

In BC, the *TP53* gene is the most frequently mutated, being present in about 30–35% of primary invasive cases [57]. *TP53* mutations vary according to BC subtypes, being mutated in 80% of triple-negative breast cancers (TNBC) and 70% of HER2-positive cases [57].

Owing to the high incidence of TNBC, *TP53* mutations constitute an important biomarker in clinical practice and a potential therapeutic target [57]. *TP53* mutation status is determined by DNA sequencing and immunohistochemistry (IHC) [55].

For a long time, *TP53-mutated* tumors were unresponsive to drugs. However, recent preclinical studies have introduced compounds capable of restoring wild p53 properties, presenting new anticancer treatment options [55,57]. Among them are COTI-2, PRIMA-1, APR-246, PK11007, and 3-quinuclidinone derivatives [57]. According to Synnott et al. [58], COTI-2 proved capable of reactivating mutant p53, inducing a therapeutic apoptotic response in TNBC cells [58]. Lee et al. [59] showed a relationship between PRIMA-1 and the expression of apoptosis proteins in MDA-231 cells with mutated p53 protein [59].

Furthermore, *TP53* has great potential as a cancer molecular risk signature, similar to *BRCA1*. Both tumor suppressor genes are potential biomarkers for surveillance, early risk assessment and predisposition to BC, being therapeutic targets for chemoprevention and targeted therapies [60].

**Figure 1:**
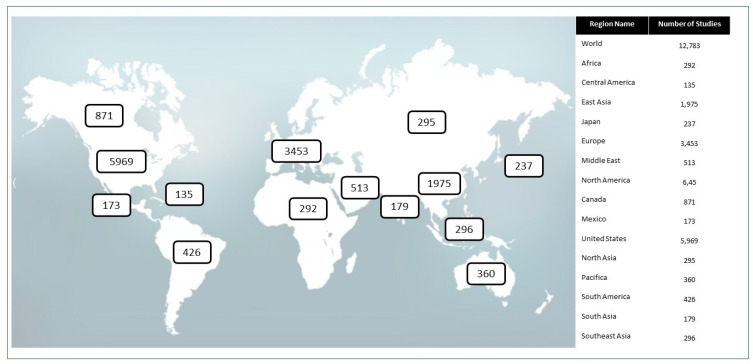
Global distribution of clinical trials using the term “Breast Cancer”. Clinical studies that have “Breast Cancer” as keywords are shown here. Studies in Europe correspond to 27.01% of all clinical trials, and United States correspond to 46.69%. Labels give the exact number of studies located in different regions. Studies with no location are not included in the counts or on the map, and studies with multiple locations are included in all corresponding regions. Adapted from: https://clinicaltrials.gov.

Breast cancer remains one of the main types of cancer investigated in clinical trials due to tumor frequency, heterogeneity, and aggressiveness. Figure 1 shows the number of clinical trials related to breast cancer worldwide.

### 3.2. Other Markers

#### 3.2.1. Genes Alterations

##### *BRCA1/BRCA2* 

*BRCA1* and *BRCA2* are tumor suppressor genes with a fundamental role in DNA repair through the homologous recombination pathway [61]. Germline mutations in these genes are associated with an increased risk of developing breast and ovarian cancer [62], with the mean cumulative breast cancer risk at 80 years of age for *BRCA1* and *BRCA2* genes being 72% and 69%, respectively [3].

Loss of function of these genes generates inefficient DNA repair, increasing mutation rates, and contributing to tumor development [61]. Patients who have pathogenic or likely pathogenic *BRCA1* variants have a predisposition to TNBC, while the presence of pathogenic or likely pathogenic variants in *BRCA2* are associated with ER-positive tumors [63]. Data on the predictive and prognostic value of *BRCA* mutations in patient survival with non-metastatic BC are conflicting [64].

Women who carry *BRCA* mutations are more likely to develop secondary cancer and bilateral mastectomy is recommended. Studies suggest that women who carry *BRCA1/BRCA2* mutations and undergo bilateral mastectomy are less likely to die from BC than women who were treated with unilateral mastectomy [65].

Tumors that have deleterious *BRCA1/BRCA2* mutations are more sensitive to DNA damaging agents, such as interchain cross-linking agents (platinum or alkylating agents), topoisomerase II inhibitors (anthracyclines), or PARP inhibitors [64]. Treatment with PARP inhibitors (olaparib and talazoparib) was approved for metastatic BC with germline pathogenic or probably pathogenic *BRCA1* or *BRCA2* variants, after evidence of longer progression-free survival, less side effects, and better quality of life compared to standard chemotherapies [63].

##### *PTEN* 

Phosphatase and tensin homolog (*PTEN*) is a tumor suppressor gene considered one of the most frequently altered genes in human cancer, including BC, and its role is intrinsically related to cell cycle progression, cell growth, and survival [66].

Tumor cells with *PTEN* deletions or mutations have significantly increased migration and invasion activity, promoting proliferation, invasion and metastasis. In metastatic BC cells, PTEN levels are much lower than in localized cancer cells [67].

The loss of function of *PTEN* leads to excessive activation of the PI3K/Akt oncogenic pathway, which stimulates cell growth and survival [68], and loss of *PTEN* activity, due to protein, genetic, or epigenetic alterations, has been reported in almost half of all BC cases [66]. *PTEN* inactivation occurs mainly due to somatic mutations [67].

Although most studies have not yet reported an association between *PTEN* loss and prognosis in BC patients enrolled in clinical trials, emerging evidence suggests that the downregulation of *PTEN* expression may be associated with worse outcomes in BC HR+/HER2− or HER2+ [66].

*PTEN* loss negatively affects sensitivity to CDK4/6 inhibitors, initiating signaling cascades that hyperactivate cyclins/CDKs, in addition to affecting the activity of *BRAF*, *EGFR* and immunological “checkpoint” inhibitors, which can be a mechanism of resistance to various treatments [69].

Some phase II and III clinical trials with translational analyses are exploring the predictive role of PTEN in response to different antitumor agents in both HER2-positive and -negative BC. However, the lack of consistency and reproducibility between clinical studies makes it difficult to interpret the real meaning of *PTEN* loss due to the heterogeneity of treatment regimens in patient cohorts [66]. Therefore, although there is some evidence of an association between *PTEN* functional status, clinical outcome, and response to various treatments, robust data are lacking to adequately establish its predictive/prognostic role in BC [66].

##### *CHEK2* 

Checkpoint Kinase 2 (*CHEK2*) gene encodes the protein serine/threonine CHK2 kinase, which is involved in DNA damage repair [70,71]. It functions as an essential tumor suppressor gene for cell cycle regulation, cell proliferation inhibition, DNA repair activation, and apoptosis [72]. Abnormal *CHEK2* expression can lead to cancer [73].

*CHEK2* germline mutations are associated with susceptibility to several types of cancer [70], with a mutation frequency of 1.08% in patients with BC [74]. *CHEK2* pathogenic variants lose protein kinase activity and confer a moderate relative risk increase (2–3) of developing BC [72]. Most patients with pathogenic or probably pathogenic variants develop BC subtypes luminal A or luminal B [74]. 

Several studies have found an association between *CHEK2* variants and ER-positive BC [75,76,77], thus the use of tamoxifen in patients with *CHEK2*-related BC is plausible [72]. CHK2 kinase domain mutations may affect cellular susceptibility to chemotherapy and induce apoptosis [78]. Although some *CHEK2* mutations have been associated with increased risk of BC and response to chemotherapy, further studies are needed to provide more accurate data [73].

##### *ATM* 

Among the most common BC susceptibility genes is the ataxia-telangiectasia mutated (*ATM*), a gene with pathogenic or probably pathogenic variants of moderate penetrance associated with the DNA double-strand break repair mechanism and which has a mutation frequency of 0.78% in patients with BC [74]. *ATM* gene expresses proteins that participate in DNA repair and cell cycle regulation, which is critical in situations of cell stress and DNA damage response [74,79,80].

*ATM* gene mutations have moderate penetrance, and heterozygous carriers have a 2–5 relative risk of developing BC, mostly hormone-receptor- or HER2-positive [79,80]. Many BC patients with *ATM* mutations develop an intermediate and high-grade disease, with higher chance of lymph node metastasis, more aggressive tumors, and worse prognosis [81].

*ATM* is an effective target for BC treatment due to its role as one of the DNA damage response junction points, which are involved in important signaling pathways, such as PI3K-AKT, MEK-ERK [82]. Gilardini et al. [83] demonstrated that reducing ATM levels in cancer cells is capable of increasing sensitivity to PARP inhibitors in BC cell lines [83]. Alterations in this gene can sensitize cancer cells to platinum-derived drugs. However, *ATM* mutations increase second tumor risk after radiotherapy [81].

##### *PALB2* 

*PALB2* (partner and localizer of *BRCA2*) is a tumor suppressor that participates in the maintenance of genome integrity. Pathogenic variants lead to a 2–30 relative risk of developing BC [84]. The BC cumulative risk in patients with a germline *PALB2* mutation up to age of 70 reaches 35%, and the 10-year survival is lower when compared to patients without *PALB2* mutations [85]. 

*PALB2* is one of eight genes frequently mutated in metastatic BC [86]. Analysis of nearly 3000 BC patients in China showed that *PALB2* pathogenic variants resulted in lower overall survival [87]. Heikkinen et al. [88] reported that *PALB2* BC patients were more likely to exhibit the triple-negative phenotype, in addition to having a more advanced disease stage, higher Ki67 levels, and lower survival [88].

Recent studies suggest that platinum-based therapeutic regimens associated with PARP inhibitors have great potential in patients with *PALB2* germline variants [89,90].

##### *BRIP1* 

*BRIP1* gene (breast cancer 1 interacting helicase 1) is necessary for DNA cross-links repair, which maintains genome stability. Mutated or overexpressed *BRIP1* is associated with BC onset and is a strong candidate for tumor progression [91,92]. *BRIP1* is located in the long arm of chromosome 17 and encodes a protein belonging to the RecQ DEAH helicase family that helps repair damaged DNA by interacting with BRCA1 [91,93,94,95]. Therefore, if BRIP1 is incomplete or lost, it does not interact with BRCA1 and cannot repair damaged DNA [91]. In this way, *BRIP1* plays a vital role in preserving cell’s genetic information and acts as a tumor suppressor [91,93,94].

*BRIP1* may be the gene involved in the onset of BC in families that do not have *BRCA1/BRCA2* mutations [95]. A recent study showed its association with rare missense *BRIP1* alleles and also two SNPs, with BC, attributing prognostic value to this gene [92,96].

*BRIP1* overexpression has been associated with breast tumor subtypes, promoter methylation status, and the survival of BC patients. These findings suggest that *BRIP1* may not only be a predictive BC development and prognosis molecular biomarker but may also function as a latent therapeutic target [91].

One of the therapeutic strategies being studied for *BRIP1* mutated tumors is the use of PARP1 inhibitors. Furthermore, BRIP1-deficient cells as well as BRCA-deficient cells are more sensitive to cisplatin treatment [97].

##### *CDH1* 

The *CDH1* gene encodes the E-cadherin cell adhesion molecule, which suppresses the spread of tumor cells (metastasis) [98]. The reduced function and expression of E-cadherin is associated with cancer metastasis due to loss of cell adhesion, resulting in increased cell motility that allows cancer cells to cross the basement membrane and invade nearby tissues [94]. Patients with *CDH1*-promoter hypermethylation have a 5.83-fold increased risk of BC [99].

*CDH1* dysfunction can lead to worse prognosis and lower survival [100]. *CDH1* hypermethylation is generally increased in HER2- and ER-negative BC, with no association with PR status [99]. Shinozaki et al. [101] demonstrated that *CDH1* was the most frequent methylated gene (90%) in cases with sentinel lymph node metastasis, supporting the associations of *CDH1* hypermethylation and metastasis [101]. Sebova et al. [102] proposed that *CDH1* hypermethylation can be used as a biomarker for tumor metastatic potential [102].

*CDH1* is a potential new drug target, and its hypermethylation can be reversed through demethylation, with the use of DNA methylation inhibitors (DNMTs), for example 5-Aza-CdR and 5-fluoro-2′-deoxycytidine which have been used in human lung cancer and BC cells, and 5-fluoro-2′-deoxycytidine is currently in clinical trials for treatment of BC and other solid tumors [99].

##### *BARD1* 

BARD1 (BRCA1-associated ring domain 1) is a BRCA-binding partner protein essential for DNA damage repair associated with BC susceptibility [103].

Interestingly, partial repression of Bard1 in mice using antisense RNAs resulted in the development of early malignancy stages phenotype in murine mammary epithelial cell lines, suggesting a role for *BARD1* in tumorigenesis [104]. Zhu et al. [105] reported that tamoxifen-resistant BC cells express significantly more BARD1 and BRCA1, leading to resistance to chemotherapy (DNA-damaging chemotherapy), including cisplatin and adriamycin, but not paclitaxel. Furthermore, higher expression of BARD1 and BRCA1 is associated with worse prognosis for patients with early BC, especially those who received radiotherapy, indicating a potential use of PI3K inhibitors to reverse chemoresistance and radioresistance in ER-positive BC patients [105].

*BARD1* may play an important role in BC pathogenesis and chemoresistance mechanisms. Some studies suggest that the role of *BARD1* in BC is mainly related to TNBC [106]. In vitro and in vivo studies indicate a potential clinical benefit of PARP inhibitors in *BARD1* mutation patients [103].

##### *PIK3CA* 

Phosphatidylinositol-3-kinase (PI3K), gene symbol (*PIK3CA*) regulates proliferation and apoptosis, and somatic mutations in *PIK3CA* can activate these processes [107]. PI3Ks are involved in several cellular processes, such as protein synthesis, cell proliferation, survival, glucose homeostasis, and DNA repair [108,109].

*PIK3CA* mutations represent one of the most common BC changes [110], and activating *PIK3CA* mutations are found in approximately 30–40% of cancer patients, inducing hyperactivation of the α-PI3K isoform [111,112]. These mutations are associated with chemoresistance and poor prognosis with reduced overall survival (19.6 months versus 23.5 months) [113,114]. Breast cancer *PIK3CA* gene mutations have been shown to be oncogenic, showing a role in tumor pathogenesis and progression [110,115].

Alpelisib is an orally available α-selective PI3K inhibitor that is 50 times more potent against α-PI3K than other isoforms [116]. Reinhardt et al. [107] demonstrated the resistance of early BC with *PIK3CA* somatic mutation to adjuvant therapy with aromatase inhibitors, suggesting tamoxifen as the preferred therapy in these patients [107].

Table 1 provide information on classical biomarkers and gene alterations discussed so far.

#### 3.2.2. MicroRNAs

MicroRNAs (miRNAs) are formed by a single-stranded non-coding RNA with 19–24 nucleotides and can often act as tumor suppressors or promoters [117,118]. miRNAs act in the regulation of cell–cell and cell–extracellular matrix adhesion molecules promoting local infiltration and dissemination of tumor cells in the systemic circulation [119] and have been suggested as possible cancer biomarkers.

According to Li et al. [120] high expression levels of miR 3662, miR-146a, and miR-1290 in exosomal miRNAs were associated with lymph node metastasis and BC stage. In patients with BC stages I, II, and III, miR-1290 expression was higher than in stage IV patients, which is evidence of it being a potential biomarker for early BC detection [120]. Savan et al. [121] showed that miRNA-10b promotes metastatic cell invasion and migration. This study shows that anti-miR10b can inhibit the miR-10b target without toxicity [121]. MiR-148a expression was associated with unfavorable clinical parameters and lower survival in BC patients and according to Li et al. (2020), MiR-148a plays an oncogenic or tumor suppressor role in different cancer types [122].

Zhang et al. [123] revealed that increased expression of miR-1246 and miR-155 in BC patients is predictive for trastuzumab therapy resistance, which is evidence of it being a clinical prognostic marker [123]. Nair et al. [119] shows the importance of miRNAs for BC diagnosis and prognosis, suggesting the use of a BC-specific miRNAs panel as a clinical assessment of microRNAs levels [119].

#### 3.2.3. uPA/PAI-1

Urokinase-type plasminogen activator (uPA) and plasminogen activator inhibitor-1 (PAI-1) are involved in hemostasis and predict impaired-BC patient survival and are used as biomarkers in cancer progression [124,125].

According to Melzer et al. [126], long-term direct coculture of human BC cells alters uPA and PAI-1 expression, favoring the spread of cancer cells and increasing metastasis [126]. Jevric et al. [127] points out that in patients with negative-lymph-node and HER2-negative BC and, *PAI-1* gene 4G/5G variants may have prognostic significance, and the subgroup of patients with homozygous recessive genotype of *PAI-1* (-675 4G/4G) may have worse disease progression when compared to patients with dominant heterozygous/homozygous genotypes (-675 4G/5G and -675 5G/5G) [127].

uPA/PAI-1 is used together with other biomarkers in routine clinical practice, guiding the therapeutic choice, but, so far, uPA/PAI-1 is not considered sufficient to replace established parameters in clinical practice [128]. Uhl et al. [125] showed that heteromerization of uPA and PAI-1 attracts neutrophils to cancerous lesions, thereby supporting tumor growth and metastasis. This study suggests that blocking uPA-PAI-1 heterodimerization by a new molecule inhibitor can prevent tumor progression in highly aggressive tumor patients with elevated uPA-PAI-1 levels [125].

Stromal co-expression of uPA and PAI-1 in BC has been associated with malignant behavior and consecutively poor outcomes in distant recurrence-free survival and overall survival [129]. In this sense, uPA-PAI-1 can be useful for prognosis and therapy response prediction [129].

#### 3.2.4. PD-1/PD-L1

Programmed death ligand 1 (PD-L1) and programmed cell death protein 1 (PD-1) receptors are related to immune control and interact by helping tumor cells escape the immune system [130]. PD-1/PD-L1 pathway regulate immune tolerance to the tumor microenvironment, being responsible for a declined immune response against tumor cells, due to inhibition of T-cell activation, proliferation, survival, and cytotoxic [131].

Tu et al. [130] showed that intracellular PD-L1 regulates the DNA damage response, acting as an RNA-binding protein and promoting the mRNA stability of *NBS1*, *BRCA1,* and other genes, thereby being a potential therapeutic target. Furthermore, the PD-L1 antibody, H1A, can induce PD-L1 degradation, aiding radiotherapy and cisplatin therapy [130].

Zhang et al. [132] showed that D-mannose can be an ally in the immune and radiotherapy of patients with triple-negative immunomodulatory metastatic BC. This study demonstrates that D-mannose reduces PD-L1 and promotes the activation of T cell and the killing of tumor cells by T cells in vitro [132]. In addition, according to Song et al. [133], a promising candidate for future clinical trials is albumin nanoparticles in combination with α-PD1, which cause the long-term remission of metastatic BC in mice [133].

#### 3.2.5. MSI

Microsatellites are DNA elements composed of short repeat sequences, and microsatellite repeat instability (MSI) is a phenotype commonly associated with the inactivation of DNA mismatch repair (MMR) genes, increasing frameshift mutations in some cancer-related genes and leading to tumor development [134].

MSI molecular diagnosis can be obtained by PCR as shown by Long et al. [135] or by DNA sequencing technologies, resulting in greater accuracy [135,136]. Klouch et al. [137] developed multiplex drop-off droplet digital PCR (ddPCR) assays, targeting four BC microsatellites and showing that MSI can be an actionable marker for immunotherapy [137]. BC diagnosis using predefined microsatellite panels is still a challenge, unlike most tumor types that need a limited number of markers to provide accurate diagnosis [135].

### 3.3. Gene Expression Profiling

Early-stage BC treatment is based on adjuvant systemic therapy (chemotherapy), endocrine therapy, agents against human epidermal growth factor receptor 2 (HER2) or a combination of these drugs [138]. In general, chemotherapy decision is based on the estimated risk of distant recurrence or metastasis [139]. However, a considerable number of patients receive chemotherapy unnecessarily, resulting in exposure to adverse effects of adjuvant therapy without a significant therapeutic gain [138].

In recent decades, advances in early detection, chemoprevention, targeted surgeries, and more effective adjuvant treatment have led to higher survival rates and reduced BC treatment morbidity [140,141]. New therapeutic approaches are currently available derived from a better understanding of cellular, molecular, and genomic properties that lead to oncogenesis [142,143]. Multigene expression assays, which are currently under development, will soon have a role in clinical practice [144,145].

Gene expression profiling is an emerging strategy aimed at risk prediction and treatment selection based on genomic information [146,147,148]. Gene expression profiling studies have distinguished at least four distinct molecular tumor types: luminal A, luminal B, HER2-enriched, and basal type [143,149,150]. Determining a tumor’s biological profile helps with treatment selection [151]. Genomic tests can predict clinical outcomes and support decision-making in clinical management regarding therapeutic strategies [144,149].

Table 2 summarizes the main tumor genomic tests currently available: Mammaprint^®^, Blueprint^®^, Oncotype DX^®^, Prosigna^®^, Endopredict^®^, and Breast Cancer Index^®^.

#### 3.3.1. MammaPrint^®^ and BluePrint^®^

MammaPrint^®^ (Agendia^©^ Inc., Irvine, CA, USA), a 70-gene signature, provides a binary classification of tumor prognosis (“high risk” or “low risk”) [152]. According to the National Institute for Health and Care Excellence (NICE) Diagnostic Guidance Guide, MammaPrint^®^ was designed to assess distant recurrence risk within 5 and 10 years and whether a person would benefit from chemotherapy [153]. MammaPrint^®^ provides further stratification into four risk subgroups: ultralow, low, high 1, and high 2, with specific prognoses and predictive outcomes [154].

This test is aimed at pre- and post-menopausal women with stage 1 or 2 breast cancer, a tumor of up to 5 cm, and lymph-node-negative (LN-) or lymph-node-positive (LN+) disease (up to three positive lymph nodes) and may be used regardless of estrogen receptor and human epidermal growth factor receptor 2 status. The NICE Diagnostic Guidance Guide does not recommend MammaPrint^®^ to guide adjuvant chemotherapy decisions for people with ER+/HER2−/LN− early BC because of the test’s cost-effectiveness [153].

BluePrint^®^ (Agendia^©^ Inc., Irvine, CA, USA) investigates the expression of 80 genes and defines breast cancer molecular subtypes (basal type, luminal type, or HER2 type), determining tumor behavior, long-term prognosis, and response to systemic therapy [154].

An independent validation study by the TRANSBIG consortium, a network of approximately 40 partners associated with the Breast International Group in 21 countries, showed that MammaPrint^®^ is capable of distinguishing patients at low risk from those at significant risk of distant recurrence and death [155]. In turn, the MINDACT trial (Microarray in Node negative Disease may Avoid ChemoTherapy), an international, prospective, randomized, phase III study, with the aim of validating performance of this RNA-based prognostic tool, provided level IA evidence for the clinical utility of MammaPrint^®^ when used in addition to the standard clinical–pathological criteria to select patients for adjuvant chemotherapy [138].

The MINDACT trial particularly evaluated the outcomes of patients who did not receive chemotherapy after been classified as “high risk” due to clinical tumor characteristics, but “low risk” by MammaPrint^®^, revealing that patients with these conditions had a 5-year distant metastasis-free survival rate of 95.1% despite being clinically classified as high risk [156]. Such results support that low risk MammaPrint^®^ patients can safely forgo chemotherapy. 

The prospective study IMPACt (Measuring the Impact of MammaPrint on Adjuvant and Neoadjuvant Treatment in Breast Cancer Patients: A Prospective Registry) recruited 452 patients to measure the effect of MammaPrint^®^ and BluePrint^®^ results in chemotherapy treatment decisions for all early-stage, ER+, and HER2− patients. According to this study, 88.5% of the treatment plans coincided with MammaPrint^®^ results, indicating that physicians can make treatment decisions in clinical practice based on MammaPrint^®^. Furthermore, in patients clinically classified as high-risk but identified as low-risk by MammaPrint^®^, there was a 60.0% reduction in treatment chemotherapy recommendations [139].

#### 3.3.2. Oncotype DX^®^

Oncotype DX^®^ (Exact Sciences Corporation^©^, Madison, WI, USA) is the most common tumor gene expression profile used in the United States [157] and its impact on treatment decisions with adjuvant chemotherapy has been evaluated by several studies [145]. Oncotype DX^®^ evaluates expression of 21 genes, of which 16 genes are related to cancer and distant recurrence-free survival, and 5 are reference genes (normalization) [153].

Oncotype DX^®^ is intended for pre- and post-menopausal women with early-stage ER+/HER2− BC and either LN− or LN+ (up to three positive lymph nodes) to predict cancer recurrence after treatment [153,158,159,160]. The test offers a recurrence score (RS), inferred by an algorithm based on the expression data of 21 genes, and guides chemoendocrine prescription for ER+/HER2− BC early treatment [144]. The RS ranges from 0 to 100 and is used to quantify recurrence risk at 10 years, assuming 5 years of endocrine therapy [153]. The current cut-off points are <18 (low risk of distant recurrence), between 18 and 30 (intermediate risk), and ≥31 (high-risk) [153].

Clinical guidelines from the American Society of Clinical Oncology (ASCO) and the National Comprehensive Cancer Network (NCCN) recommend the use of Oncotype DX^®^ in specific situations [157]. Its use has successfully reduced the prescription of systemic chemotherapy for patients with low or intermediate RS values [158,159,161]. In a recent meta-analysis study, Davey et al. [145] demonstrated that Oncotype DX^®^ appears to be effective in estimating the locoregional recurrence risk in early-stage ER+/HER2− BC [145].

The clinical use of Oncotype DX^®^ in early-stage ER+/HER2− disease facilitates the customization of combined chemoendocrine therapy for patients at higher risk of recurrence [144], avoiding overtreatment for those who will not benefit from these therapies [158,159].

#### 3.3.3. Prosigna^®^ (PAM50)

Prosigna^®^ Breast Cancer Assay (PAM50) (Veracyte^©^ Inc., South San Francisco, CA, USA) defines the risk group (low, intermediate, or high), recurrence risk, and intrinsic tumor subtype (luminal A, luminal B, HER2-enriched, or basal type). The test is designed to provide information about BC subtype and predict 10-year distant recurrence-free survival. It is indicated for post-menopausal patients with ER+/HER2− and lymph-node-negative (LN−) early breast cancer or lymph-node-positive (LN+), limited to three LN+ [153].

PAM50 measures the expression of fifty genes used for subtyping, eight internal genes used for signal normalization, six for positive controls, and eight for negative controls [153]. With these data, Prosigna^®^ ranks the risk of distant recurrence within 10 years, assuming 5 years of endocrine therapy, based on the PAM50 gene signature and clinicopathologic features, such as tumor size, nodal status, and proliferation score. The proliferation score is determined by evaluating multiple genes associated with the proliferation pathway [153]. 

PAM 50 gives a score between 0 and 100. Based on this score and nodal status, samples are classified into risk categories: 1) LN-negative—low risk (0 to 40), intermediate risk (41 to 60), or high risk (61 to 100); and 2) LN-positive (up to three positive lymph nodes)—low risk (0 to 15), intermediate risk (16 to 40), or high risk (41 to 100) [153].

According to the Clinical Practice Guidelines of the European Society for Medical Oncology (ESMO), PAM-50 is recognized with an evidence level of 1B. ESMO guidelines point out that gene expression panels, such as Breast Cancer Index^®^, EPclin, MammaPrint^®^, Oncotype DX^®^ and Prosigna^®^ (PAM50), can be used to obtain additional prognostic and/or predictive information to complement the pathology assessment and predict the benefit of adjuvant chemotherapy [162].

#### 3.3.4. EndoPredict

The Endopredict^®^ Assay (Myriad Genetics^©^ Inc., Salt Lake City, UT, USA) is a breast cancer prognostic test designed for determination of the 10-year risk of distant recurrence (metastatic disease), the probability of distant recurrence 5–15 years after diagnosis, and the estimated absolute benefit of chemotherapy at 10 years [163]. This information can guide therapeutic decisions by identifying which patients are at a sufficiently low risk of distant recurrence and can safely forego chemotherapy, and which patients are at high risk of distant recurrence and may need adjuvant chemotherapy in addition to endocrine therapy [164,165,166].

Endopredict^®^ is indicated for pre- and post-menopausal women with early breast cancer that is ER+, HER2−, LN− or LN+ type (up to three positive lymph nodes) [153]. The test is intended for in vitro analysis performed on formalin-fixed, paraffin-embedded (FFPE) tumor tissue and biopsy specimens of primary invasive tumors [163]. The analysis is performed using real-time quantitative polymerase chain reaction (qRT-PCR) [167] and investigates RNA expression of twelve genes: three genes associated with proliferation, five genes associated with hormone receptor, three reference genes (normalization), and one control gene [153].

Based on molecular data obtained by qRT-PCR, the Endopredict^®^ Score (EP) is inferred. An EP score of 0 to <5 indicates a low risk of distant disease recurrence in the next 10 years, whereas an EP score of 5 to 15 indicates a high risk [153]. EP is combined with clinical tumor characteristics (size and nodal status) to result in a comprehensive risk score, the EPclin [167]. The EPclin score estimates the probability of developing metastases within 10 years, assuming 5 years of endocrine therapy. An EPclin score <3.3 indicates a low risk of metastases (less than 10%), whereas scores >3.3 indicate a high risk of a less favorable clinical outcome. The EPclin risk score is a more significant predictor of the 10-year risk of distant recurrence than the molecular score alone [163].

Dubsky et al. [168] assessed the clinical relevance of EPclin by comparing its risk rating with the rating assigned by three well-established guidelines or recommendations: NCCN 2007 Guidelines, German S3 Guidelines 2008, and St. Gallen Consensus Recommendations 2011 [168]. The authors demonstrated that 58–61% of women classified as high and intermediate risk according to clinical guidelines were reclassified as low risk through EPclin, and that there was a 5% rate of distant metastasis at 10 years among these patients. Data suggest that Endopredict^®^ may contribute to a reduction in chemotherapy indications for ER+ tumors in post-menopausal women with a limited number of clinical risk factors [168].

The prediction potential for distant recurrence of EndoPredict^®^ was validated in prospective–retrospective studies in three different cohorts of phase III trials [164,168,169,170,171]. According to biomarker guidelines [172], Endopredict^®^ received a level of evidence score of 1B obtained from retrospective analyses of data from prospective studies on the prognostic value of the test in ER+ breast cancer [167,173]. EndoPredict^®^ was incorporated into ASCO^®^ Practice Guidelines [174] and into NCCN Clinical Practice Guidelines in Oncology [175].

#### 3.3.5. Breast Cancer Index^®^

The Breast Cancer Index^®^ (BCI) assay (Biotheranostics^©^, Inc., San Diego, CA, USA) is an algorithmic signature based on gene expression and is composed of two panels of independent functional biomarkers: the Molecular Grade Index (MGI) with five genes (*BUB1B*, *CENPA*, *NEK2*, *RACGAP1* and *RRM2*), which evaluates tumor proliferation, and the expression ratio of *HOXB13*/*IL17BR* (H/I) genes, which evaluates estrogen signaling [167,176,177].

The combination of MGI and H/I parameters provides a BCI prognostic score capable of measuring general (0 to 10 years) and late (5 to 10 years) distance recurrence risk [178,179,180]. The risk of relapse is a constant concern for patients with HR+ breast cancer, and approximately half of disease recurrences occur after five years of adjuvant antiestrogen therapy [180]. The H/I ratio is the predictive component of the BCI signature and has been shown to be effective in predicting endocrine response in different therapeutic scenarios with consistent predictive evidence in at least five studies [176,178,179,180,181].

Zhang et al. [180] examined the prognostic performance of BCI through retrospective analyses of tumor samples from patients treated with tamoxifen from a prospective randomized study (Stockholm TAM, *n* = 317) [182] and from an institutional multi-component cohort (*n* = 358) [177,180,183]. The study revealed that for the Stockholm TAM cohort, BCI stratified the majority (65%) of patients as low risk, with less than 3% distant recurrence rates 0–5 years and 5–10 years. The multi-institutional cohort had major tumors, and the BCI classified 55% of patients as being low risk, with less than 5% distant recurrence rate for 0–5 years and 5–10 years. The data support the hypothesis that BCI has prognostic sustainability to assess early and late distant recurrence risk [180].

Bartlett et al. [176] evaluated BCI for its ability to predict the benefit of extended endocrine therapy. The study was conducted in patients previously randomized to Adjuvant Tamoxifen—To Offer More? (aTTom Trial), a multi-institutional, prospective–retrospective study with tumor blocks available in FFPE. The study revealed that a high expression of H/I in the BCI test was predictive of an endocrine response and identified a subset of HR+/LN+ patients who would receive significant benefit from 10 versus 5 years of tamoxifen therapy. The results obtained lead to an evidence level of 1B for the BCI test as a predictive biomarker of benefit from extended endocrine therapy. BCI can be used in clinical routine in post-menopausal patients with ER+/HER2-, and LN− or LN+ tumors (up to three positive lymph nodes) who are on antiestrogen therapies, including tamoxifen and aromatase inhibitors [160,176].

BCI is currently recognized by the NCCN and ASCO^®^ as the only genomic test capable of predicting the benefit of extended endocrine therapy in early-stage HR+ breast cancer [175,184]. In other words, BCI has the potential to prevent overtreatment of patients for whom endocrine therapy beyond 5 years is unlikely to result in benefit [185,186,187,188]. Therefore, predictive endocrine response biomarkers, such as BCI can significantly improve patient selection for prolonged therapy [176].

## 4. Single-Cell Approach in Breast Cancer

Breast cancers are still classified based on tumor structure and cell morphology and subcategorized according to hormone receptors, protein levels or specific genetic alterations [189]. Single-cell-based genomic technologies and in situ spatial multiplexing methods provide a more integrated and highly enriching view regarding therapeutic personalization and the discovery of more accurate and predictive potential biomarkers [190,191].

New initiatives in the area of single cells have been useful for elucidating cell heterogeneity, tissue architecture in a transcriptional atlas (robust cell taxonomy, cell spatial map, and ecotype clusters), and cell landscape [191,192]. New insights into cell biology, disease etiology, drug response, molecular resolution, disease taxonomy, the identification of heterotypic cell interactions, and the determination of cell differentiation events in breast cancer are now achievable [190].

Fathi et al. [193] highlight the use of single-cell integrated profiling of extracellular vesicle secretions and cell transcriptomes as sources of biomarkers for aggressive metastatic breast cancer [193]. Cani et al. [194] add the importance of circulating tumor cells and tumor DNA as information sources processed through single-cells, which is capable of providing serial noninvasive monitoring of the evolving tumor genome and informing actionable predictive biomarkers for precisely guided treatment in metastatic breast cancer [194].

Single-cell studies have provided an understanding of the tumor microenvironment and its relationship with breast cancer evolution, discovering new signatures, such as subpopulations of cancer-associated fibroblasts (CAFs) that are highly specific to tumor stage [195], the epigenetic mechanisms of resistance [196,197], potential biomarkers, and optimal combination strategies of immune therapy in a multi-omic view [198,199,200,201,202].

The integration of single-cell in basic research has led to improvements in personalized therapy by identifying potential treatment targets for the development of new drugs and revealing promising biomarkers to monitor treatment efficacy and guide therapeutic decision-making [198,203,204].

## 5. New Potential Biomarkers in PGCCs: Future Perspectives

Polyploidy or whole-genome duplication (WGD) results in long-term actions in evolution (organism) and ontogenesis (somatics), being a source of increased organismal complexity and evolutionary plasticity to compensate for cell proliferation, stress, and specific functional load [205]. Polyploidy increases stress adaptation [206]. Although, such cells are subject to slower proliferation, genome instability, high energy cost, and mitotic defects that result in some detrimental effects, this trait regulates numerous biological pathways, operating through genetic and epigenetic mechanisms, network adaptive responses, self-organization, phylogenetic regression between multicellularity, and unicellularity and aneuploidies [205,206,207,208].

Casotti et al. [209] provided an integrative literature review about the subject, showing computational applications for understanding cancer polyploidy and how PGCCs contribute to tumorigenesis [209]. Polyploid giant cancer cells (PGCCs) are capable of dynamically restructuring the genome, epigenome (epigenetic rearrangements), and tumor microenvironment [210,211,212].

Table 3 highlights the potential biomarkers for PGCCs in different tumors.

**Figure 2:**
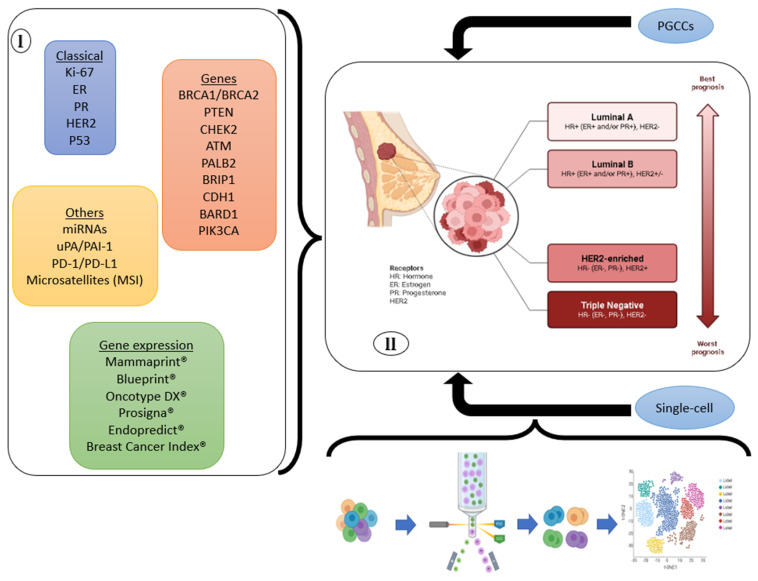
Biomarkers correlated with breast cancer subtypes and future perspectives. (**I**) Recent studies highlight new, and more predictive and accurate biomarkers that are capable of personalizing breast cancer treatment and diagnosis. Consequently, (**II**) there is a precise direction in terms of classification of classical breast cancer subtypes. Furthermore, new classifications become possible through single-cell strategies and analysis of PGCCs.

Figure 2 summarizes the biomarkers presented here, the characteristics of the breast cancer subtypes, and the main tumor genomic tests currently available.

## 6. Conclusions

Advances in molecular research in recent years allowed for a better knowledge of tumor characteristics, broadening our understanding about breast cancer biomarkers, and improving individualized patient therapy. In this review, we summarize the available traditional, novel, and potential biomarkers, also including gene expression profiling, breast cancer single-cell, and polyploid giant cancer cells.

## Figures and Tables

**Table 1:** Reported biomarkers data sources.

Biomarker	Information in Breast Cancer	Reference
Ki-67	Protein expression is related to cell proliferation and higher protein levels to biological aggressiveness.	Menon et al. [30] and Rakha [31]
ER	Nuclear receptor that acts as a ligand-activated transcription factor. The main isoform is ERα that is associated with cell survival and proliferation.	Fuentes and Silveyra [37] and Mills et al. [39]
PR	Nuclear receptor that acts as a ligand-activated transcription factor. It is associated with the expression of genes related to the cell cycle, cell differentiation, and proliferation.	Hilton et al. [33] and Cenciarini and Proietti [44]
HER2	Receptor signaling leads to tumor growth and proliferation, adhesion, cell survival, and metastasis.	Harbeck et al. [3] and Nicolini et al. [40]
p53	Tumor suppressor protein involved in cell cycle arrest, differentiation, senescence, apoptosis, cell growth, and DNA repair. Its degradation is linked to tumor formation, progression, and metastasis.	Shahbandi et al. [55] and Xu et al. [56]
*BRCA1/* *BRCA2*	Tumor suppressor genes fundamental to DNA repair. Loss of function generates inefficient DNA repair, increasing mutation rates, and contributing to tumor development.	Ayed-Guerfali et al. [61]
*PTEN*	Tumor suppressor gene related to cell cycle progression, cell growth, and survival. Deletions or mutations are related to proliferation, invasion, and metastasis.	Carbognin et al. [66] and Chen et al. [67]
*CHEK2*	Tumor suppressor gene related to cell cycle regulation, inhibition of cell proliferation, activation of DNA repair, and apoptosis. It encodes the protein serine/threonine CHK2 kinase, which is involved in DNA damage repair.	Kleiblova et al. [70], Boonen et al. [71], and Greville-Heygate et al. [72]
*ATM*	Gene associated with the DNA double-strand break repair mechanism. It encodes proteins that participate in DNA repair and cell cycle regulation.	Toss et al. [74], Cunha et al. [79], and Moslemi et al. [80]
*PALB2*	Tumor suppressor gene that encodes PALB2, responsible for BRCA2 nuclear localization and DNA damage repair.	Nepomuceno et al. [84]
*BRIP1*	Tumor suppressor gene that encodes a protein belonging to the RecQ DEAH helicase family that helps repair damaged DNA by interacting with BRCA1.	Khan et al. [91] and Moyer et al. [92]
*CDH1*	Tumor suppressor gene that encodes the E-cadherin cell–cell adhesion protein, that prevents migration of tumor cells, avoiding cancer progression and metastases.	Bücker and Lehmann [98]
BARD1	BRCA1-binding partner protein that is related to DNA damage repair. Higher expression is associated with worse prognosis.	Zheng et al. [103] and Zhu et al. [105]
*PIK3CA*	Gene involved in regulation of proliferation and apoptosis. PI3K protein is involved in several cellular processes, such as protein synthesis, cell proliferation, survival, glucose homeostasis, and DNA repair.	Reinhardt et al. [107], Thorpe et al. [108], and Venetis et al. [109]

ER: estrogen receptor; PR: progesterone receptor.

**Table 2:** Characteristics of Gene Expression Profile Tests for breast cancer.

	Number of Genes	Information Provided	Indication
**MammaPrint^®^** **/BluePrint^®^**	70/80	Risk of distant recurrence (5 and 10 years), with or without benefit of chemotherapy	Pre- and post-menopausal
			Early stage
			Tumor up to ~5 cm
		Identification of intrinsic molecular subtypes, information about tumor behavior, long-term prognosis and response to systemic therapy	ER+/ER−
			HER2+/HER2−
			LN− or LN+ (up to 3 LN+)
**Oncotype DX^®^**	21	Prediction of cancer recurrence in 10 years assuming 5 years of endocrine therapy	Pre- and post-menopausal
			Early stage
			ER+
			HER2−
			LN− or LN+ (up to 3 LN+)
**Prosigna^®^**	50	Cancer subtype, risk stratification, prediction of 10 years distant recurrence-free survival assuming 5 years of endocrine therapy	Post-menopausal
			Early stage
			ER+
			HER2−
			LN− or LN+ (up to 3 LN+)
**Endopredict^®^**	12	Risk of distant recurrence (10 years), probability of distant recurrence (5–15 years) after diagnosis, estimated absolute benefit of chemotherapy at 10 years	Pre- and post-menopausal
			Early stage
			ER+
			HER2−
			LN− or LN+ (up to 3 LN+)
**Breast Cancer Index^®^**	7	Risk of general (0 to 10 years) and late (5 to 10 years) distant recurrence, predicts benefit of extended endocrine therapy in early stage HR+ breast cancer	Post-menopausal
			Early stage
			ER+
			HER2−
			LN− or LN+ (up to 3 LN+)

ER: estrogen receptor; LN: lymph node; HR: hormone receptor.

**Table 3:** Potential biomarkers for PGCCs.

References	Potential Biomarkers	Type of Tumor
Herbein [213], Song et al. [214], and Zhang et al. [215]	Myc, PI3K, Akt, p53, Rb, and IL-6—elevated Myc expression, activation of the PI3K/Akt pathway, repression of p53 and Rb genes, and loss of Rb leading to increased IL-6 production, correlating with the appearance of PGCCs.	Cancer in general
	CD44, CD133, OCT4, SOX2, and Nanog—high expression in PGCCs reveals the potential for multidifferentiation and self-renewal capacity similar to embryonic stem cells, with such expression pattern transmissible to the produced diploid descendant cells.	
	S100A4—expression is correlated with the invasive and metastatic ability of PGCCs and their progeny cells.	
	ZEB1—increased expression in prostate cancer PGCCs.	
	Twist, Slug, and Snail—expression buildup in colon cancer PGCCs.	
	N-cadherin, vimentin, and cathepsin—increased expression in PGCCs of some cancer cell lines.	
	SPO11 and Mos-kinase—meiotic and telomere-related mechanisms may play a role in PGCC neosis.	
	ASAH1—elevated expression in radiation-induced PGCC in prostate cancer and lung cancer.	
	AURK—correlated with induction of polyploid cells by regulating mitosis or the arrest of cell division.	
	CSC, CD44, and CD133—expression of markers of stem cell properties in PGCCs.	
	GCM1/syncytin-1—highlight the presence of cell fusion during the formation of PGCCs.	
El Baba et al. [216] and Nehme et al. [217]	Ki67—a proliferative index marker, which is also strongly linked to tumor initiation, growth, and metastasis, able to evaluate the proliferation of PGCCs that acquired embryonic-like stemness and a hybrid epithelial–mesenchymal phenotype.	Breast cancer
	EZH2, SUZ12, and Myc—PGCCs show an overexpression of these proteins.	
	Ki67, Vimentin, CD49f, CD44, CD24, OCT4, NANOG, and E-cadherin—call attention to the high expression when associated with PGCCs.	
	EpCAM—highlighted a downregulation in PGCCs.	
Liu et al. [218] and Liu et al. [219]	CDC25C—Regulation of its expression and subcellular localization correlates with the formation of PGCCs by activating cyclin B1–CDK1.	Breast and ovarian cancer
	p38MAPK-ERK-JNK—Cell cycle progression and formation of PGCCs by regulation of CDC25C.	
Tagal and Roth [220] and Zhang et al. [221]	Aurora A and B—Inhibition induces PGCC formation.	Breast and lung cancer
Bowers et al. [222], Niu et al. [223], Silva et al. [224], and Yart et al. [225]	p21—marks a temporary arrest in the cell cycle of PGCCs similar to senescence.	Ovarian cancer
	LCB-II and p62/SQSTM1—autophagy markers with increased levels, but a low autophagic flow for PGCCs is highlighted, while their derived progeny has high rates of autophagy during neosis.	
	TNF-α, NF-κB, lipopolysaccharide (LPS), and IL-6—more regulated in PGCCs, while pathways related to cell proliferation and division were inhibited. IL-6 facilitates PGCC formation and embryonic stem acquisition via an autocrine loop. PGCCs can use IL-6 protein as a paracrine mechanism to facilitate the transformation of fibroblasts into more tumor-promoting CAFs for chemoresistance.	
	PAX 8, WT-1, Ki-67, ER, and p53—positive and aberrant expression (overexpression) correlated with the presence of PGCCs.	
	GRP78—its overexpression is correlated with the UPR (unfolded protein response) activation marker aspect. UPR induces ovarian cancer cell fusion and the formation of PGCCs.	
Thura et al. [226]	PRL3—induces the formation of PGCCs that express markers of embryonic stem cells, such as SOX2 and OCT4.	Ovarian cancer, melanoma and stomach cancer
Fu et al. [227], Li et al. [228], Peerpen et al. [229], and Zhao et al. [230]	PLK4—kinase overexpressed in PGCCs, and descendant cells showed strong migration and invasion abilities. Its interaction with CDC25C is associated with the formation of PGCCs.	Colorectal cancer
	GCM1, Syncytin-1, ASCT-2, OCT-4, NANOG, CD44, and CD133—correlated with the formation of PGCCs via GCM1-mediated cell fusion, regulating syncytin-1 expression, and generating offspring expressing embryonic stem cell markers, including and with increased expression of epithelial-to-mesenchymal transition (EMT) markers.	
	ARID1A—its inhibition correlated with the increase in PGCCs and multicellular spheroids.	
	S100A10, CD44, and CD133—correlated with expression and nuclear localization, modified by SUMOylation, with high proliferation and migration of PGCCs and their daughter cells (with stem cell properties), and with differentiation, metastases, and recurrences by regulation of the expression of ARHGEF18, PTPRN2, and DEFA3.	
Liu et al. [231] and You et al. [232]	Cyclin B1, CDC25C, CDK1, E-cadherin, and EIF-4A—demonstrated lower expression in PGCCs when compared to normal cancer cells.	Head and neck cancer
	Vimentin and CD133—demonstrated increased expression in PGCCs.	
	RIPK1—its overexpression was induced through the AMPK-mTOR pathway, which promoted the formation of PGCCs upon analysis of the transcriptional and epigenetic landscape of these cells.	
Lu et al. [233] and White-Gilbertson et al. [234]	ASAH1—interferes with generation of PGCC offspring.	Melanoma and prostate cancer
	p53—inhibition promotes generation of PGCC.	
	INSIG1—has lower expression in PGCCs and acts by negatively regulating cholesterol metabolism.	
	SR-B1—is a type 1 class B scavenger receptor that presents high levels of expression in PGCCs.	
Pustovalova et al. [235]	p53, OCT4 (low expression), NANOG (low expression), CD44 (present expression), CD133 (present expression), and p21—ionizing radiation influences the expression and accumulation of these proteins in a quiescent state (dormancy) and spontaneous formation of PGCCs with or without slow cycling, followed by re-entry into the cell cycle and formation of therapy-resistant clones with increased migratory and invasive activity.	Lung cancer
Voelkel-Johnson [236]	YAP—associated with ASAH1 promotes the formation of PGCC progeny.	Liver cancer

PGCCs: polyploid giant cancer cells; ER: estrogen receptor.

## Data Availability

Not applicable.

## References

[1] Schick J., Ritchie R.P., Restini C. (2021). Breast Cancer Therapeutics and Biomarkers: Past, Present, and Future Approaches. Breast Cancer.

[2] Stickeler E. (2011). Prognostic and Predictive Markers for Treatment Decisions in Early Breast Cancer. Breast Care.

[3] Harbeck N., Penault-Llorca F., Cortes J., Gnant M., Houssami N., Poortmans P., Ruddy K., Tsang J., Cardoso F. (2019). Breast cancer. Nat. Rev. Dis. Primers.

[4] Barzaman K., Karami J., Zarei Z., Hosseinzadeh A., Kazemi M.H., Moradi-Kalbolandi S., Safari E., Farahmand L. (2020). Breast cancer: Biology, biomarkers, and treatments. Int. Immunopharmacol..

[5] Mehta S., Shelling A., Muthukaruppan A., Lasham A., Blenkiron C., Laking G., Print C. (2010). Predictive and prognostic molecular markers for cancer medicine. Ther. Adv. Med. Oncol..

[6] Wu H.J., Chu P.Y. (2021). Recent Discoveries of Macromolecule- and Cell-Based Biomarkers and Therapeutic Implications in Breast Cancer. Int. J. Mol. Sci..

[7] Taneja P., Maglic D., Kai F., Zhu S., Kendig R.D., Fry E.A., Inoue K. (2010). Classical and Novel Prognostic Markers for Breast Cancer and their Clinical Significance. Clin. Med. Insights Oncol..

[8] Rakha E.A., Reis-Filho J.S., Baehner F., Dabbs D.J., Decker T., Eusebi V., Fox S.B., Ichihara S., Jacquemier J., Lakhani S.R. (2010). Breast cancer prognostic classification in the molecular era: The role of histological grade. Breast Cancer Res..

[9] Veronesi U., Galimberti V., Zurrida S., Merson M., Greco M., Luini A. (1993). Prognostic significance of number and level of axillary node metastases in breast cancer. Breast.

[10] Chung H.L., Le-Petross H.T., Leung J.W.T. (2021). Imaging Updates to Breast Cancer Lymph Node Management. Radiographics.

[11] Slanetz P.J., Moy L., Baron P., diFlorio R.M., Green E.D., Heller S.L., Holbrook A.I., Lee S.J., Lewin A.A., Lourenco A.P. (2017). ACR Appropriateness Criteria® Monitoring Response to Neoadjuvant Systemic Therapy for Breast Cancer. J. Am. Coll. Radiol..

[12] Carter C.L., Allen C., Henson D.E. (1989). Relation of tumor size, lymph node status, and survival in 24,740 breast cancer cases. Cancer.

[13] Mikami Y., Yamada A., Suzuki C., Adachi S., Harada F., Yamamoto S., Shimada K., Sugae S., Narui K., Chishima T. (2021). Predicting Nonsentinel Lymph Node Metastasis in Breast Cancer: A Multicenter Retrospective Study. J. Surg. Res..

[14] Jozsa F., Ahmed M., Baker R., Douek M. (2019). Is sentinel node biopsy necessary in the radiologically negative axilla in breast cancer?. Breast Cancer Res. Treat..

[15] Anderson T.L., Glazebrook K.N., Murphy B.L., Viers L.D., Hieken T.J. (2017). Cross-sectional imaging to evaluate the extent of regional nodal disease in breast cancer patients undergoing neoadjuvant systemic therapy. Eur. J. Radiol..

[16] Foulkes W.D., Reis-Filho J.S., Narod S.A. (2010). Tumor size and survival in breast cancer--a reappraisal. Nat Rev. Clin. Oncol..

[17] Cuesta Cuesta A.B., Ríos M.D.M., Meseguer M.R.N., Velasco J.A.G., Martínez M.M., Sotillos S.B., Griego E.A. (2019). Accuracy of tumor size measurements performed by magnetic resonance, ultrasound and mammography, and their correlation with pathological size in primary breast cancer. Cir. Esp. (Engl. Ed.).

[18] Hata T., Takahashi H., Watanabe K., Takahashi M., Taguchi K., Itoh T., Todo S. (2004). Magnetic resonance imaging for preoperative evaluation of breast cancer: A comparative study with mammography and ultrasonography. J. Am. Coll. Surg..

[19] Hlawatsch A., Teifke A., Schmidt M., Thelen M. (2002). Preoperative assessment of breast cancer: Sonography versus MR imaging. AJR Am. J. Roentgenol..

[20] Kneeshaw P.J., Turnbull L.W., Smith A., Drew P.J. (2003). Dynamic contrast enhanced magnetic resonance imaging aids the surgical management of invasive lobular breast cancer. Eur. J. Surg. Oncol..

[21] Haraldsdóttir K.H., Jónsson Þ., Halldórsdóttir A.B., Tranberg K.G., Ásgeirsson K.S. (2017). Tumor Size of Invasive Breast Cancer on Magnetic Resonance Imaging and Conventional Imaging (Mammogram/Ultrasound): Comparison with Pathological Size and Clinical Implications. Scand. J. Surg..

[22] Berg W.A., Gutierrez L., NessAiver M.S., Carter W.B., Bhargavan M., Lewis R.S., Ioffe O.B. (2004). Diagnostic accuracy of mammography, clinical examination, US, and MR imaging in preoperative assessment of breast cancer. Radiology.

[23] Gu J., Groot G., Boden C., Busch A., Holtslander L., Lim H. (2018). Review of Factors Influencing Women’s Choice of Mastectomy Versus Breast Conserving Therapy in Early Stage Breast Cancer: A Systematic Review. Clin. Breast Cancer.

[24] Gu J., Delisle M., Engler-Stringer R., Groot G. (2019). Mastectomy versus breast-conservation therapy: An examination of how individual, clinicopathologic, and physician factors influence decision-making. Curr. Oncol..

[25] Elston C.W., Ellis I.O. (1991). Pathological prognostic factors in breast cancer. I. The value of histological grade in breast cancer: Experience from a large study with long-term follow-up. Histopathology.

[26] Rakha E.A., El-Sayed M.E., Lee A.H., Elston C.W., Grainge M.J., Hodi Z., Blamey R.W., Ellis I.O. (2008). Prognostic significance of Nottingham histologic grade in invasive breast carcinoma. J. Clin. Oncol..

[27] Wang M., Klevebring D., Lindberg J., Czene K., Grönberg H., Rantalainen M. (2016). Determining breast cancer histological grade from RNA-sequencing data. Breast Cancer Res..

[28] Sotiriou C., Wirapati P., Loi S., Harris A., Fox S., Smeds J., Nordgren H., Farmer P., Praz V., Haibe-Kains B. (2006). Gene expression profiling in breast cancer: Understanding the molecular basis of histologic grade to improve prognosis. J. Natl. Cancer Inst..

[29] Ivshina A.V., George J., Senko O., Mow B., Putti T.C., Smeds J., Lindahl T., Pawitan Y., Hall P., Nordgren H. (2006). Genetic reclassification of histologic grade delineates new clinical subtypes of breast cancer. Cancer Res..

[30] Menon S.S., Guruvayoorappan C., Sakthivel K.M., Rasmi R.R. (2019). Ki-67 protein as a tumour proliferation marker. Clin. Chim. Acta.

[31] Rakha E.A., Chmielik E., Schmitt F.C., Tan P.H., Quinn C.M., Gallagy G. (2022). Assessment of Predictive Biomarkers in Breast Cancer: Challenges and Updates. Pathobiology.

[32] Remnant L., Kochanova N.Y., Reid C., Cisneros-Soberanis F., Earnshaw W.C. (2021). The intrinsically disorderly story of Ki-67. Open Biol..

[33] Hilton H.N., Clarke C.L., Graham J.D. (2018). Estrogen and progesterone signalling in the normal breast and its implications for cancer development. Mol. Cell Endocrinol..

[34] Zhang A., Wang X., Fan C., Mao X. (2021). The Role of Ki67 in Evaluating Neoadjuvant Endocrine Therapy of Hormone Receptor-Positive Breast Cancer. Front. Endocrinol..

[35] Yeo B., Dowsett M. (2015). Neoadjuvant endocrine therapy: Patient selection, treatment duration and surrogate endpoints. Breast.

[36] Polley M.Y., Leung S.C., McShane L.M., Gao D., Hugh J.C., Mastropasqua M.G., Viale G., Zabaglo L.A., Penault-Llorca F., Bartlett J.M. (2013). An international Ki67 reproducibility study. J. Natl. Cancer Inst..

[37] Fuentes N., Silveyra P. (2019). Estrogen receptor signaling mechanisms. Adv. Protein Chem. Struct. Biol..

[38] Hernando C., Ortega-Morillo B., Tapia M., Moragón S., Martínez M.T., Eroles P., Garrido-Cano I., Adam-Artigues A., Lluch A., Bermejo B. (2021). Oral Selective Estrogen Receptor Degraders (SERDs) as a Novel Breast Cancer Therapy: Present and Future from a Clinical Perspective. Int. J. Mol. Sci..

[39] Mills J.N., Rutkovsky A.C., Giordano A. (2018). Mechanisms of resistance in estrogen receptor positive breast cancer: Overcoming resistance to tamoxifen/aromatase inhibitors. Curr. Opin. Pharmacol..

[40] Nicolini A., Ferrari P., Duffy M.J. (2018). Prognostic and predictive biomarkers in breast cancer: Past, present and future. Semin. Cancer Biol..

[41] Hanker A.B., Sudhan D.R., Arteaga C.L. (2020). Overcoming Endocrine Resistance in Breast Cancer. Cancer Cell.

[42] Brufsky A.M., Dickler M.N. (2018). Estrogen Receptor-Positive Breast Cancer: Exploiting Signaling Pathways Implicated in Endocrine Resistance. Oncologist.

[43] Brett J.O., Spring L.M., Bardia A., Wander S.A. (2021). ESR1 mutation as an emerging clinical biomarker in metastatic hormone receptor-positive breast cancer. Breast Cancer Res..

[44] Cenciarini M.E., Proietti C.J. (2019). Molecular mechanisms underlying progesterone receptor action in breast cancer: Insights into cell proliferation and stem cell regulation. Steroids.

[45] Mote P.A., Gompel A., Howe C., Hilton H.N., Sestak I., Cuzick J., Dowsett M., Hugol D., Forgez P., Byth K. (2015). Progesterone receptor A predominance is a discriminator of benefit from endocrine therapy in the ATAC trial. Breast Cancer Res. Treat..

[46] Li Z., Wei H., Li S., Wu P., Mao X. (2022). The Role of Progesterone Receptors in Breast Cancer. Drug Des. Devel. Ther..

[47] Mohammed H., Russell I.A., Stark R., Rueda O.M., Hickey T.E., Tarulli G.A., Serandour A.A., Birrell S.N., Bruna A., Saadi A. (2015). Progesterone receptor modulates ERα action in breast cancer. Nature.

[48] Islam M.S., Afrin S., Jones S.I., Segars J. (2020). Selective Progesterone Receptor Modulators-Mechanisms and Therapeutic Utility. Endocr. Rev..

[49] Lamb C.A., Fabris V.T., Lanari C. (2020). Progesterone and breast. Best Pract. Res. Clin. Obstet. Gynaecol..

[50] Gaddy V.T., Barrett J.T., Delk J.N., Kallab A.M., Porter A.G., Schoenlein P.V. (2004). Mifepristone induces growth arrest, caspase activation, and apoptosis of estrogen receptor-expressing, antiestrogen-resistant breast cancer cells. Clin. Cancer Res..

[51] Meira D.D., Casotti M.C., Braga R.F.R., Filho L.C.G.S., Guimarães A.P., Campanharo C.V., Duque D.A., Barbosa D.G., Lopes L.M., Kohls V.N.G., Hu S. (2023). Chapter 28—Targeted cancer therapy: The future of drug combinations. Breaking Tolerance to Antibody-Mediated Immunotherapy, Novel Sensitizing Agents for Therapeutic Anti-EGFR Antibodies.

[52] Chung I., Reichelt M., Shao L., Akita R.W., Koeppen H., Rangel L., Schaefer G., Mellma I., Sliwkowski M.X. (2016). High cell-surface density of HER2 deforms cell membranes. Nat. Commun..

[53] Wynn C.S., Tang S.C. (2022). Anti-HER2 therapy in metastatic breast cancer: Many choices and future directions. Cancer Metastasis Rev..

[54] Kunte S., Abraham J., Montero A.J. (2020). Novel HER2-targeted therapies for HER2-positive metastatic breast cancer. Cancer.

[55] Shahbandi A., Nguyen H.D., Jackson J.G. (2020). TP53 Mutations and Outcomes in Breast Cancer: Reading beyond the Headlines. Trends Cancer.

[56] Xu Z., Wu W., Yan H., Hu Y., He Q., Luo P. (2021). Regulation of p53 stability as a therapeutic strategy for cancer. Biochem. Pharmacol..

[57] Duffy M.J., Synnott N.C., Crown J. (2018). Mutant p53 in breast cancer: Potential as a therapeutic target and biomarker. Breast Cancer Res. Treat..

[58] Synnott N.C., O’Connell D., Crown J., Duffy M.J. (2020). COTI-2 reactivates mutant p53 and inhibits growth of triple-negative breast cancer cells. Breast Cancer Res. Treat..

[59] Lee K., Wang T., Paszczynski A.J., Daoud S.S. (2006). Expression proteomics to p53 mutation reactivation with PRIMA-1 in breast cancer cells. Biochem. Biophys. Res. Commun..

[60] Raimundo L., Ramos H., Loureiro J.B., Calheiros J., Saraiva L. (2020). BRCA1/P53: Two strengths in cancer chemoprevention. Biochim. Biophys. Acta Rev. Cancer.

[61] Ayed-Guerfali D.B., Kridis-Rejab W.B., Ammous-Boukhris N., Ayadi W., Charfi S., Khanfir A., Sellami-Boudawara T., Frikha M., Daoud J., Mokdad-Gargouri R. (2021). Novel and recurrent BRCA1/BRCA2 germline mutations in patients with breast/ovarian cancer: A series from the south of Tunisia. J. Transl. Med..

[62] Liu L., Matsunaga Y., Tsurutani J., Akashi-Tanaka S., Masuda H., Ide Y., Hashimoto R., Inuzuka M., Watanabe C., Taruno K. (2020). BRCAness as a prognostic indicator in patients with early breast cancer. Sci. Rep..

[63] Tutt A.N.J., Garber J.E., Kaufman B., Viale G., Fumagalli D., Rastogi P., Gelber R.D., de Azambuja E., Fielding A., Balmaña J. (2021). Adjuvant Olaparib for Patients with *BRCA1*- or *BRCA2*-Mutated Breast Cancer. N. Engl. J. Med..

[64] De Talhouet S., Peron J., Vuilleumier A., Friedlaender A., Viassolo V., Ayme A., Bodmer A., Treilleux I., Lang N., Tille J.C. (2020). Clinical outcome of breast cancer in carriers of BRCA1 and BRCA2 mutations according to molecular subtypes. Sci. Rep..

[65] Godet I., Gilkes D.M. (2017). BRCA1 and BRCA2 mutations and treatment strategies for breast cancer. Integr. Cancer Sci. Ther..

[66] Carbognin L., Miglietta F., Paris I., Dieci M.V. (2019). Prognostic and Predictive Implications of PTEN in Breast Cancer: Unfulfilled Promises but Intriguing Perspectives. Cancers.

[67] Chen J., Sun J., Wang Q., Du Y., Cheng J., Yi J., Xie B., Jin S., Chen G., Wang L. (2022). Systemic Deficiency of PTEN Accelerates Breast Cancer Growth and Metastasis. Front. Oncol..

[68] Xie P., Peng Z., Chen Y., Li H., Du M., Tan Y., Zhang X., Lu Z., Cui C.P., Liu C.H. (2021). Neddylation of PTEN regulates its nuclear import and promotes tumor development. Cell Res..

[69] Costa C., Wang Y., Ly A., Hosono Y., Murchie E., Walmsley C.S., Huynh T., Healy C., Peterson R., Yanase S. (2020). PTEN Loss Mediates Clinical Cross-Resistance to CDK4/6 and PI3Kα Inhibitors in Breast Cancer. Cancer Discov..

[70] Kleiblova P., Stolarova L., Krizova K., Lhota F., Hojny J., Zemankova P., Havranek O., Vocka M., Cerna M., Lhotova K. (2019). Identification of deleterious germline CHEK2 mutations and their association with breast and ovarian cancer. Int. J. Cancer.

[71] Boonen R.A.C.M., Wiegant W.W., Celosse N., Vroling B., Heijl S., Kote-Jarai Z., Mijuskovic M., Cristea S., Solleveld-Westerink N., van Wezel T. (2022). Functional Analysis Identifies Damaging CHEK2 Missense Variants Associated with Increased Cancer Risk. Cancer Res..

[72] Greville-Heygate S.L., Maishman T., Tapper W.J., Cutress R.I., Copson E., Dunning A.M., Haywood L., Jones L.J., Eccles D.M. (2020). Pathogenic Variants in *CHEK2* Are Associated With an Adverse Prognosis in Symptomatic Early-Onset Breast Cancer. JCO Precis. Oncol..

[73] Apostolou P., Papasotiriou I. (2017). Current perspectives on CHEK2 mutations in breast cancer. Breast Cancer.

[74] Toss A., Tenedini E., Piombino C., Venturelli M., Marchi I., Gasparini E., Barbieri E., Razzaboni E., Domati F., Caggia F. (2021). Clinicopathologic Profile of Breast Cancer in Germline ATM and CHEK2 Mutation Carriers. Genes.

[75] Decker B., Allen J., Luccarini C., Pooley K.A., Shah M., Bolla M.K., Wang Q., Ahmed S., Baynes C., Conroy D.M. (2017). Rare, protein-truncating variants in *ATM*, *CHEK2* and *PALB2*, but not *XRCC2*, are associated with increased breast cancer risks. J. Med. Genet..

[76] Cybulski C., Wokołorczyk D., Jakubowska A., Huzarski T., Byrski T., Gronwald J., Masojć B., Deebniak T., Górski B., Blecharz P. (2011). Risk of breast cancer in women with a CHEK2 mutation with and without a family history of breast cancer. J. Clin. Oncol..

[77] Weischer M., Nordestgaard B.G., Pharoah P., Bolla M.K., Nevanlinna H., Van’t Veer L.J., Garcia-Closas M., Hopper J.L., Hall P., Andrulis I.L. (2012). CHEK2*1100delC heterozygosity in women with breast cancer associated with early death, breast cancer-specific death, and increased risk of a second breast cancer. J. Clin. Oncol..

[78] Ansari N., Shahrabi S., Khosravi A., Shirzad R., Rezaeean H. (2019). Prognostic Significance of CHEK2 Mutation in Progression of Breast Cancer. Lab. Med..

[79] Cunha R., Nejo P., Bento S., Vaz F. (2021). *ATM* germline variants and male breast cancer. BMJ Case Rep..

[80] Moslemi M., Moradi Y., Dehghanbanadaki H., Afkhami H., Khaledi M., Sedighimehr N., Fathi J., Sohrabi E. (2021). The association between ATM variants and risk of breast cancer: A systematic review and meta-analysis. BMC Cancer.

[81] Stucci L.S., Internò V., Tucci M., Perrone M., Mannavola F., Palmirotta R., Porta C. (2021). The ATM Gene in Breast Cancer: Its Relevance in Clinical Practice. Genes.

[82] Mirza-Aghazadeh-Attari M., Recio M.J., Darband S.G., Kaviani M., Safa A., Mihanfar A., Sadighparvar S., Karimian A., Alemi F., Majidinia M. (2021). DNA damage response and breast cancer development: Possible therapeutic applications of ATR, ATM, PARP, BRCA1 inhibition. DNA Repair..

[83] Montani M.S.G., Prodosmo A., Stagni V., Merli D., Monteonofrio L., Gatti V., Gentileschi M.P., Barilà D., Soddu S. (2013). ATM-depletion in breast cancer cells confers sensitivity to PARP inhibition. J. Exp. Clin. Cancer Res..

[84] Nepomuceno T.C., De Gregoriis G., de Oliveira F.M.B., Suarez-Kurtz G., Monteiro A.N., Carvalho M.A. (2017). The Role of PALB2 in the DNA Damage Response and Cancer Predisposition. Int. J. Mol. Sci..

[85] Li A., Geyer F.C., Blecua P., Lee J.Y., Selenica P., Brown D.N., Pareja F., Lee S.S.K., Kumar R., Rivera B. (2019). Homologous recombination DNA repair defects in *PALB2*-associated breast cancers. NPJ Breast Cancer.

[86] Lefebvre C., Bachelot T., Filleron T., Pedrero M., Campone M., Soria J.C., Massard C., Lévy C., Arnedos M., Lacroix-Triki M. (2016). Mutational Profile of Metastatic Breast Cancers: A Retrospective Analysis. PLoS Med..

[87] Deng M., Chen H.H., Zhu X., Luo M., Zhang K., Xu C.J., Hu K.M., Cheng P., Zhou J.J., Zheng S. (2019). Prevalence and clinical outcomes of germline mutations in BRCA1/2 and PALB2 genes in 2769 unselected breast cancer patients in China. Int. J. Cancer.

[88] Heikkinen T., Kärkkäinen H., Aaltonen K., Milne R.L., Heikkilä P., Aittomäki K., Blomqvist C., Nevanlinna H. (2009). The breast cancer susceptibility mutation PALB2 1592delT is associated with an aggressive tumor phenotype. Clin. Cancer Res..

[89] Basourakos S.P., Li L., Aparicio A.M., Corn P.G., Kim J., Thompson T.C. (2017). Combination Platinum-based and DNA Damage Response-targeting Cancer Therapy: Evolution and Future Directions. Curr. Med. Chem..

[90] Tischkowitz M., Balmaña J., Foulkes W.D., James P., Ngeow J., Schmutzler R., Voian N., Wick M.J., Stewart D.R., Pal T. (2021). Management of individuals with germline variants in PALB2: A clinical practice resource of the American College of Medical Genetics and Genomics (ACMG). Genet. Med..

[91] Khan U., Khan M.S. (2021). Prognostic Value Estimation of BRIP1 in Breast Cancer by Exploiting Transcriptomics Data Through Bioinformatics Approaches. Bioinform. Biol. Insights.

[92] Moyer C.L., Ivanovich J., Gillespie J.L., Doberstein R., Radke M.R., Richardson M.E., Kaufmann S.H., Swisher E.M., Goodfellow P.J. (2020). Rare *BRIP1* Missense Alleles Confer Risk for Ovarian and Breast Cancer. Cancer Res..

[93] Sato K., Koyasu M., Nomura S., Sato Y., Kita M., Ashihara Y., Adachi Y., Ohno S., Iwase T., Kitagawa D. (2017). Mutation status of RAD51C, PALB2 and BRIP1 in 100 Japanese familial breast cancer cases without BRCA1 and BRCA2 mutations. Cancer Sci..

[94] Sheikh A., Hussain S.A., Ghori Q., Naeem N., Fazil A., Giri S., Sathian B., Mainali P., Al Tamimi D.M. (2015). The spectrum of genetic mutations in breast cancer. Asian Pac. J. Cancer Prev..

[95] Ouhtit A., Gupta I., Shaikh Z. (2016). BRIP1, a potential candidate gene in development of non-BRCA1/2 breast cancer. Front. Biosci..

[96] Li X., Li Z., Yang M., Luo Y., Hu L., Xiao Z., Huang A., Huang J. (2021). Two tSNPs in BRIP1 are associated with breast cancer during TDT analysis. Mol. Genet. Genomic. Med..

[97] Cantor S.B., Guillemette S. (2011). Hereditary breast cancer and the BRCA1-associated FANCJ/BACH1/BRIP1. Future Oncol..

[98] Bücker L., Lehmann U. (2022). CDH1 (*E-cadherin*) Gene Methylation in Human Breast Cancer: Critical Appraisal of a Long and Twisted Story. Cancers.

[99] Huang R., Ding P., Yang F. (2015). Clinicopathological significance and potential drug target of CDH1 in breast cancer: A meta-analysis and literature review. Drug Des. Devel. Ther..

[100] Corso G., Figueiredo J., De Angelis S.P., Corso F., Girardi A., Pereira J., Seruca R., Bonanni B., Carneiro P., Pravettoni G. (2020). E-cadherin deregulation in breast cancer. J. Cell. Mol. Med..

[101] Shinozaki M., Hoon D.S., Giuliano A.E., Hansen N.M., Wang H.J., Turner R., Taback B. (2005). Distinct hypermethylation profile of primary breast cancer is associated with sentinel lymph node metastasis. Clin. Cancer Res..

[102] Sebova K., Zmetakova I., Bella V., Kajo K., Stankovicova I., Kajabova V., Krivulcik T., Lasabova Z., Tomka M., Galbavy S. (2011). RASSF1A and CDH1 hypermethylation as potential epimarkers in breast cancer. Cancer Biomark..

[103] Zheng Y., Li B., Pan D., Cao J., Zhang J., Wang X., Li X., Hou W., Bao D., Ren L. (2021). Functional consequences of a rare missense BARD1 c.403G>A germline mutation identified in a triple-negative breast cancer patient. Breast Cancer Res..

[104] Irminger-Finger I., Soriano J.V., Vaudan G., Montesano R., Sappino A.P. (1998). In vitro repression of Brca1-associated RING domain gene, Bard1, induces phenotypic changes in mammary epithelial cells. J. Cell Biol..

[105] Zhu Y., Liu Y., Zhang C., Chu J., Wu Y., Li Y., Liu J., Li Q., Li S., Shi Q. (2018). Tamoxifen-resistant breast cancer cells are resistant to DNA-damaging chemotherapy because of upregulated BARD1 and BRCA1. Nat. Commun..

[106] Śniadecki M., Brzeziński M., Darecka K., Klasa-Mazurkiewicz D., Poniewierza P., Krzeszowiec M., Kmieć N., Wydra D. (2020). BARD1 and Breast Cancer: The Possibility of Creating Screening Tests and New Preventive and Therapeutic Pathways for Predisposed Women. Genes.

[107] Reinhardt K., Stückrath K., Hartung C., Kaufhold S., Uleer C., Hanf V., Lantzsch T., Peschel S., John J., Pöhler M. (2022). *PIK3CA*-mutations in breast cancer. Breast Cancer Res. Treat..

[108] Thorpe L.M., Yuzugullu H., Zhao J.J. (2015). PI3K in cancer: Divergent roles of isoforms, modes of activation and therapeutic targeting. Nat. Rev. Cancer.

[109] Venetis K., Sajjadi E., Haricharan S., Fusco N. (2020). Mismatch repair testing in breast cancer: The path to tumor-specific immuno-oncology biomarkers. Transl. Cancer Res..

[110] Zardavas D., Phillips W.A., Loi S. (2014). PIK3CA mutations in breast cancer: Reconciling findings from preclinical and clinical data. Breast Cancer Res..

[111] Anderson E.J., Mollon L.E., Dean J.L., Warholak T.L., Aizer A., Platt E.A., Tang D.H., Davis L.E. (2020). A Systematic Review of the Prevalence and Diagnostic Workup of PIK3CA Mutations in HR+/HER2- Metastatic Breast Cancer. Int. J. Breast Cancer.

[112] Goncalves M.D., Hopkins B.D., Cantley L.C. (2018). Phosphatidylinositol 3-Kinase, Growth Disorders, and Cancer. N. Engl. J. Med..

[113] Mosele F., Stefanovska B., Lusque A., Tran Dien A., Garberis I., Droin N., Le Tourneau C., Sablin M.P., Lacroix L., Enrico D. (2020). Outcome and molecular landscape of patients with PIK3CA-mutated metastatic breast cancer. Ann. Oncol..

[114] Sobhani N., Roviello G., Corona S.P., Scaltriti M., Ianza A., Bortul M., Zanconati F., Generali D. (2018). The prognostic value of PI3K mutational status in breast cancer: A meta-analysis. J. Cell Biochem..

[115] Lian J., Xu E.W., Xi Y.F., Wang H.W., Bu P., Wang J.F., Wang L.X. (2020). Clinical-Pathologic Analysis of Breast Cancer With PIK3CA Mutations in Chinese Women. Technol. Cancer Res. Treat..

[116] André F., Ciruelos E.M., Juric D., Loibl S., Campone M., Mayer I.A., Rubovszky G., Yamashita T., Kaufman B., Lu Y.S. (2021). Alpelisib plus fulvestrant for PIK3CA-mutated, hormone receptor-positive, human epidermal growth factor receptor-2-negative advanced breast cancer: Final overall survival results from SOLAR-1. Ann. Oncol..

[117] Lu T.X., Rothenberg M.E. (2018). MicroRNA. J. Allergy Clin. Immunol..

[118] Soleimanpour E., Babaei E., Hosseinpour-Feizi M.A., Montazeri V. (2019). Circulating miR-21 and miR-155 as potential noninvasive biomarkers in Iranian Azeri patients with breast carcinoma. J. Cancer Res. Ther..

[119] Nair M.G., Somashekaraiah V.M., Ramamurthy V., Prabhu J.S., Sridhar T.S. (2021). miRNAs: Critical mediators of breast cancer metastatic programming. Exp. Cell Res..

[120] Li S., Zhang M., Xu F., Wang Y., Leng D. (2021). Detection significance of miR-3662, miR-146a, and miR-1290 in serum exosomes of breast cancer patients. J. Cancer Res. Ther..

[121] Savan N.A., Saavedra P.V., Halim A., Yuzbasiyan-Gurkan V., Wang P., Yoo B., Kiupel M., Sempere L., Medarova Z., Moore A. (2022). Case report: MicroRNA-10b as a therapeutic target in feline metastatic mammary carcinoma and its implications for human clinical trials. Front. Oncol..

[122] Li D., Wang J., Ma L.J., Yang H.B., Jing J.F., Jia M.M., Zhang X.J., Guo F., Gao J.N. (2020). Identification of serum exosomal miR-148a as a novel prognostic biomarker for breast cancer. Eur. Rev. Med. Pharmacol. Sci..

[123] Zhang Z., Zhang L., Yu G., Sun Z., Wang T., Tian X., Duan X., Zhang C. (2020). Exosomal miR-1246 and miR-155 as predictive and prognostic biomarkers for trastuzumab-based therapy resistance in HER2-positive breast cancer. Cancer Chemother. Pharmacol..

[124] Märkl B., Kazik M., Harbeck N., Jakubowicz E., Hoffmann R., Jung T., Steinfeld D., Schenkirsch G., Schlimok G., Oruzio D. (2019). Impact of uPA/PAI-1 and disseminated cytokeratin-positive cells in breast cancer. BMC Cancer.

[125] Uhl B., Mittmann L.A., Dominik J., Hennel R., Smiljanov B., Haring F., Schaubächer J.B., Braun C., Padovan L., Pick R. (2021). uPA-PAI-1 heteromerization promotes breast cancer progression by attracting tumorigenic neutrophils. EMBO Mol. Med..

[126] Melzer C., von der Ohe J., Otterbein H., Ungefroren H., Hass R. (2019). Changes in uPA, PAI-1, and TGF-β Production during Breast Cancer Cell Interaction with Human Mesenchymal Stroma/Stem-Like Cells (MSC). Int. J. Mol. Sci..

[127] Jevrić M., Matić I.Z., Krivokuća A., Crnogorac M.D., Besu I., Damjanović A., Branković-Magić M., Milovanović Z., Gavrilović D., Susnjar S. (2019). Association of uPA and PAI-1 tumor levels and 4G/5G variants of PAI-1 gene with disease outcome in luminal HER2-negative node-negative breast cancer patients treated with adjuvant endocrine therapy. BMC Cancer.

[128] Reix N., Lodi M., Jankowski S., Molière S., Luporsi E., Leblanc S., Scheer L., Ibnouhsein I., Benabu J.C., Gabriele V. (2019). A novel machine learning-derived decision tree including uPA/PAI-1 for breast cancer care. Clin. Chem. Lab. Med..

[129] Singer C.F., Filipits M., Jahn S.W., Abete L., Jakesz R., Greil R., Bauernhofer T., Kwasny W., Seifert M., Fitzal F. (2019). Stromal coexpression of uPA/PAI-1 protein predicts poor disease outcome in endocrine-treated postmenopausal patients with receptor-positive early breast cancer. Breast.

[130] Tu X., Qin B., Zhang Y., Zhang C., Kahila M., Nowsheen S., Yin P., Yuan J., Pei H., Li H. (2019). PD-L1 (B7-H1) Competes with the RNA Exosome to Regulate the DNA Damage Response and Can Be Targeted to Sensitize to Radiation or Chemotherapy. Mol. Cell.

[131] Han Y., Liu D., Li L. (2020). PD-1/PD-L1 pathway: Current researches in cancer. Am. J. Cancer Res..

[132] Zhang R., Yang Y., Dong W., Lin M., He J., Zhang X., Tian T., Yang Y., Chen K., Lei Q.Y. (2022). D-mannose facilitates immunotherapy and radiotherapy of triple-negative breast cancer via degradation of PD-L1. Proc. Natl. Acad. Sci. USA.

[133] Song Y., Bugada L., Li R., Hu H., Zhang L., Li C., Yuan H., Rajanayake K.K., Truchan N.A., Wen F. (2022). Albumin nanoparticle containing a PI3Kγ inhibitor and paclitaxel in combination with α-PD1 induces tumor remission of breast cancer in mice. Sci. Transl. Med..

[134] Yamamoto H., Watanabe Y., Maehata T., Imai K., Itoh F. (2020). Microsatellite instability in cancer: A novel landscape for diagnostic and therapeutic approach. Arch. Toxicol..

[135] Long D.R., Waalkes A., Panicker V.P., Hause R.J., Salipante S.J. (2020). Identifying Optimal Loci for the Molecular Diagnosis of Microsatellite Instability. Clin. Chem..

[136] Hause R.J., Pritchard C.C., Shendure J., Salipante S.J. (2016). Classification and characterization of microsatellite instability across 18 cancer types. Nat. Med..

[137] Klouch K.Z., Stern M.H., Trabelsi-Grati O., Kiavue N., Cabel L., Silveira A.B., Hego C., Rampanou A., Popova T., Bataillon G. (2022). Microsatellite instability detection in breast cancer using drop-off droplet digital PCR. Oncogene.

[138] Cardoso F., van’t Veer L.J., Bogaerts J., Slaets L., Viale G., Delaloge S., Pierga J.Y., Brain E., Causeret S., DeLorenzi M. (2016). 70-Gene Signature as an Aid to Treatment Decisions in Early-Stage Breast Cancer. N. Engl. J. Med..

[139] Soliman H., Shah V., Srkalovic G., Mahtani R., Levine E., Mavromatis B., Srinivasiah J., Kassar M., Gabordi R., Qamar R. (2020). MammaPrint guides treatment decisions in breast Cancer: Results of the IMPACt trial. BMC Cancer.

[140] Rutgers E., Piccart-Gebhart M.J., Bogaerts J., Delaloge S., Veer L.V., Rubio I.T., Viale G., Thompson A.M., Passalacqua R., Nitz U. (2011). The EORTC 10041/BIG 03-04 MINDACT trial is feasible: Results of the pilot phase. Eur. J. Cancer..

[141] Keelan S., Flanagan M., Hill A.D.K. (2021). Evolving Trends in Surgical Management of Breast Cancer: An Analysis of 30 Years of Practice Changing Papers. Front. Oncol..

[142] Perou C.M., Sørlie T., Eisen M.B., van de Rijn M., Jeffrey S.S., Rees C.A., Pollack J.R., Ross D.T., Johnsen H., Akslen L.A. (2000). Molecular portraits of human breast tumours. Nature.

[143] Sørlie T., Perou C.M., Tibshirani R., Aas T., Geisler S., Johnsen H., Hastie T., Eisen M.B., van de Rijn M., Jeffrey S.S. (2001). Gene expression patterns of breast carcinomas distinguish tumor subclasses with clinical implications. Proc. Natl. Acad. Sci. USA.

[144] Paik S., Shak S., Tang G., Kim C., Baker J., Cronin M., Baehner F.L., Walker M.G., Watson D., Park T. (2004). A multigene assay to predict recurrence of tamoxifen-treated, node-negative breast cancer. N. Engl. J. Med..

[145] Davey M.G., Cleere E.F., O’Donnell J.P., Gaisor S., Lowery A.J., Kerin M.J. (2022). Value of the 21-gene expression assay in predicting locoregional recurrence rates in estrogen receptor-positive breast cancer: A systematic review and network meta-analysis. Breast Cancer Res. Treat..

[146] Albain K.S., Paik S., van’t Veer L. (2009). Prediction of adjuvant chemotherapy benefit in endocrine responsive, early breast cancer using multigene assays. Breast.

[147] Arpino G., Generali D., Sapino A., Del Matro L., Frassoldati A., de Laurentis M., Pronzato P., Mustacchi G., Cazzaniga M., De Placido S. (2013). Gene expression profiling in breast cancer: A clinical perspective. Breast.

[148] Marchionni L., Wilson R.F., Wolff A.C., Marinopoulos S., Parmigiani G., Bass E.B., Goodman S.N. (2008). Systematic review: Gene expression profiling assays in early-stage breast cancer. Ann. Intern. Med..

[149] Sotiriou C., Pusztai L. (2009). Gene-expression signatures in breast cancer. N. Engl. J. Med..

[150] Goldhirsch A., Winer E.P., Coates A.S., Gelber R.D., Piccart-Gebhart M., Thürlimann B., Senn H.J. (2013). Personalizing the treatment of women with early breast cancer: Highlights of the St Gallen International Expert Consensus on the Primary Therapy of Early Breast Cancer 2013. Ann. Oncol..

[151] Viale G., de Snoo F.A., Slaets L., Bogaerts J., van ‘t Veer L., Rutgers E.J., Piccart-Gebhart M.J., Stork-Sloots L., Glas A., Russo L. (2018). Immunohistochemical versus molecular (BluePrint and MammaPrint) subtyping of breast carcinoma. Outcome results from the EORTC 10041/BIG 3-04 MINDACT trial. Breast Cancer Res. Treat..

[152] van de Vijver M.J., He Y.D., van’t Veer L.J., Dai H., Hart A.A., Voskuil D.W., Schreiber G.J., Peterse J.L., Roberts C., Marton M.J. (2002). A gene-expression signature as a predictor of survival in breast cancer. N. Engl. J. Med..

[153] Harnan S., Tappenden P., Cooper K., Stevens J., Bessey A., Rafia R., Ward S., Wong R., Stein R.C., Brown J. (2019). Tumour profiling tests to guide adjuvant chemotherapy decisions in early breast cancer: A systematic review and economic analysis. Health Technol. Assess..

[154] Haan J.C., Bhaskaran R., Ellappalayam A., Bijl Y., Griffioen C.J., Lujinovic E., Audeh W.M., Penault-Llorca F., Mittempergher L., Glas A.M. (2022). MammaPrint and BluePrint comprehensively capture the cancer hallmarks in early-stage breast cancer patients. Genes Chromosomes Cancer.

[155] Buyse M., Loi S., van’t Veer L., Viale G., Delorenzi M., Glas A.M., d’Assignies M.S., Bergh J., Lidereau R., Ellis P. (2006). Validation and clinical utility of a 70-gene prognostic signature for women with node-negative breast cancer. J. Natl. Cancer Inst..

[156] Piccart M., van ‘t Veer L.J., Poncet C., Cardozo J.M.N.L., Delaloge S., Pierga J.Y., Vuylsteke P., Brain E., Vrijaldenhoven S., Neijenhuis P.A. (2021). 70-gene signature as an aid for treatment decisions in early breast cancer: Updated results of the phase 3 randomised MINDACT trial with an exploratory analysis by age. Lancet Oncol..

[157] Carlson J.J., Roth J.A. (2013). The impact of the Oncotype Dx breast cancer assay in clinical practice: A systematic review and meta-analysis. Breast Cancer Res. Treat..

[158] Sparano J.A., Gray R.J., Makower D.F., Pritchard K.I., Albain K.S., Hayes D.F., Jr C.E.G., Dees E.C., Goetz M.P., Olson J.A. (2018). Adjuvant Chemotherapy Guided by a 21-Gene Expression Assay in Breast Cancer. N. Engl. J. Med..

[159] Kalinsky K., Barlow W.E., Meric-Bernstam F., Gralow J.R., Albain K.S., Hayes D., Lin N., Perez E.A., Goldstein L.J., Chia S. (2021). Abstract GS3-00: First results from a phase III randomized clinical trial of standard adjuvant endocrine therapy (ET) +/− chemotherapy (CT) in patients (pts) with 1–3 positive nodes, hormone receptor-positive (HR+) and HER2-negative (HER2-) breast cancer (BC) with recurrence score (RS) < 25: SWOG S1007 (RxPonder). Cancer Res..

[160] Varnier R., Sajous C., de Talhouet S., Smentek C., Péron J., You B., Reverdy T., Freyer G. (2021). Using Breast Cancer Gene Expression Signatures in Clinical Practice: Unsolved Issues, Ongoing Trials and Future Perspectives. Cancers.

[161] McVeigh T.P., Hughes L.M., Miller N., Sheehan M., Keane M., Sweeney K.J., Kerin M.J. (2014). The impact of Oncotype DX testing on breast cancer management and chemotherapy prescribing patterns in a tertiary referral centre. Eur. J. Cancer.

[162] Duffy M.J., Harbeck N., Nap M., Molina R., Nicolini A., Senkus E., Cardoso F. (2017). Clinical use of biomarkers in breast cancer: Updated guidelines from the European Group on Tumor Markers (EGTM). Eur. J. Cancer.

[163] Myriad Genetic Laboratories, Inc. Myriad EndoPredict® Technical Specifications (21 December 2021). https://myriad.com/genetic-tests/endopredict-breast-cancer-prognostic-test/.

[164] Buus R., Sestak I., Kronenwett R., Denkert C., Dubsky P., Krappmann K., Scheer M., Petry C., Cuzick J., Dowsett M. (2016). Comparison of EndoPredict and EPclin With Oncotype DX Recurrence Score for Prediction of Risk of Distant Recurrence After Endocrine Therapy. J. Natl. Cancer Inst..

[165] Kronenwett R., Bohmann K., Prinzler J., Sinn B.V., Haufe F., Roth C., Averdick M., Ropers T., Windbergs C., Brase J.C. (2012). Decentral gene expression analysis: Analytical validation of the Endopredict genomic multianalyte breast cancer prognosis test. BMC Cancer..

[166] Müller B.M., Keil E., Lehmann A., Winzer K.J., Richter-Ehrenstein C., Prinzler J., Bangemann N., Reles A., Stadie S., Schoenegg W. (2013). The EndoPredict Gene-Expression Assay in Clinical Practice—Performance and Impact on Clinical Decisions. PLoS ONE.

[167] Harbeck N., Sotlar K., Wuerstlein R., Doisneau-Sixou S. (2014). Molecular and protein markers for clinical decision making in breast cancer: Today and tomorrow. Cancer Treat. Rev..

[168] Dubsky P., Brase J.C., Jakesz R., Rudas M., Singer C.F., Greil R., Dietze O., Luisser I., Klug E., Sedivy R. (2013). The EndoPredict score provides prognostic information on late distant metastases in ER+/HER2- breast cancer patients. Br. J. Cancer.

[169] Dubsky P., Filipits M., Jakesz R., Rudas M., Singer C.F., Greil R., Dietze O., Luisser I., Klug E., Sedivy R. (2013). EndoPredict improves the prognostic classification derived from common clinical guidelines in ER-positive, HER2-negative early breast cancer. Ann. Oncol..

[170] Filipits M., Rudas M., Jakesz R., Dubsky P., Fitzal F., Singer C.F., Dietze O., Greil R., Jelen A., Sevelda P. (2011). A new molecular predictor of distant recurrence in ER-positive, HER2-negative breast cancer adds independent information to conventional clinical risk factors. Clin. Cancer Res..

[171] Sestak I., Buus R., Cuzick J., Dubsky P., Kronenwett R., Denkert C., Ferree S., Sgroi D., Schnabel C., Baehner F.L. (2018). Comparison of the Performance of 6 Prognostic Signatures for Estrogen Receptor-Positive Breast Cancer: A Secondary Analysis of a Randomized Clinical Trial. JAMA Oncol..

[172] Simon R.M., Paik S., Hayes D.F. (2009). Use of archived specimens in evaluation of prognostic and predictive biomarkers. J. Natl. Cancer Inst..

[173] Warf M.B., Rajamani S., Krappmann K., Doedt J., Cassiano J., Brown K., Reid J.E., Kronenwett R., Roa B.B. (2017). Analytical validation of a 12-gene molecular test for the prediction of distant recurrence in breast cancer. Future Sci. OA.

[174] Harris L.N., Ismaila N., McShane L.M., Andre F., Collyar D.E., Gonzalez-Angulo A.M., Hammond E.H., Kuderer N.M., Liu M.C., Mennel R.G. (2016). Use of Biomarkers to Guide Decisions on Adjuvant Systemic Therapy for Women With Early-Stage Invasive Breast Cancer: American Society of Clinical Oncology Clinical Practice Guideline. J. Clin. Oncol..

[175] National Comprehensive Cancer Network NCCN Clinical Practice Guidelines in Oncology (NCCN Guidelines®). https://www.nccn.org/professionals/physician_gls/pdf/breast.pdf.

[176] Bartlett J.M.S., Sgroi D.C., Treuner K., Zhang Y., Ahmed I., Piper T., Salunga R., Brachtel E.F., Pirrie S.J., Schnabel C.A. (2019). Breast Cancer Index and prediction of benefit from extended endocrine therapy in breast cancer patients treated in the Adjuvant Tamoxifen-To Offer More? (aTTom) trial. Ann. Oncol..

[177] Jankowitz R.C., Cooper K., Erlander M.G., Ma X.J., Kesty N.C., Li H., Chivukula M., Brufsky A. (2011). Prognostic utility of the breast cancer index and comparison to Adjuvant! Online in a clinical case series of early breast cancer. Breast Cancer Res..

[178] Sgroi D.C., Carney E., Zarrella E., Steffel L., Binns S.N., Finkelstein D.M., Szymonifka J., Bhan A.K., Shepherd L.E., Zhang Y. (2013). Prediction of late disease recurrence and extended adjuvant letrozole benefit by the HOXB13/IL17BR biomarker. J. Natl. Cancer Inst..

[179] Sgroi D.C., Sestak I., Cuzick J., Zhang Y., Schnabel C.A., Schroeder B., Erlander M.G., Dunbier A., Sidhu K., Lopez-Knowles E. (2013). Prediction of late distant recurrence in patients with oestrogen-receptor-positive breast cancer: A prospective comparison of the breast-cancer index (BCI) assay, 21-gene recurrence score, and IHC4 in the TransATAC study population. Lancet Oncol..

[180] Zhang Y., Schnabel C.A., Schroeder B.E., Jerevall P.L., Jankowitz R.C., Fornander T., Stål O., Brufsky A.M., Sgroi D., Erlander M.G. (2013). Breast cancer index identifies early-stage estrogen receptor-positive breast cancer patients at risk for early- and late-distant recurrence. Clin. Cancer Res..

[181] Noordhoek I., Treuner K., Putter H., Zhang Y., Wong J., Meershoek-Klein Kranenbarg E., Duijm-de Carpentier M., van de Velde C.J.H., Schnabel C.A., Liefers G.J. (2021). Breast Cancer Index Predicts Extended Endocrine Benefit to Individualize Selection of Patients with HR+ Early-stage Breast Cancer for 10 Years of Endocrine Therapy. Clin. Cancer Res..

[182] Rutqvist L.E., Johansson H. (2007). Long-term follow-up of the randomized Stockholm trial on adjuvant tamoxifen among postmenopausal patients with early stage breast cancer. Acta Oncol..

[183] Ma X.J., Salunga R., Dahiya S., Wang W., Carney E., Durbecq V., Harris A., Goss P., Sotiriou C., Erlander M. (2008). A five-gene molecular grade index and HOXB13:IL17BR are complementary prognostic factors in early stage breast cancer. Clin. Cancer Res..

[184] Andre F., Ismaila N., Allison K.H., Barlow W.E., Collyar D.E., Damodaran S., Henry N.L., Jhaveri K., Kalinsky K., Kuderer N.M. (2022). Biomarkers for Adjuvant Endocrine and Chemotherapy in Early-Stage Breast Cancer: ASCO Guideline Update. J. Clin. Oncol..

[185] Davies C., Pan H., Godwin J., Gray R., Arriagada R., Raina V., Abraham M., Medeiros Alencar V.H., Badran A., Bonfill X. (2013). Long-term effects of continuing adjuvant tamoxifen to 10 years versus stopping at 5 years after diagnosis of oestrogen receptor-positive breast cancer: ATLAS, a randomised trial. Lancet.

[186] Goss P.E., Ingle J.N., Martino S., Robert N.J., Muss H.B., Piccart M.J., Castiglione M., Tu D., Shepherd L.E., Pritchard K.I. (2005). Randomized trial of letrozole following tamoxifen as extended adjuvant therapy in receptor-positive breast cancer: Updated findings from NCIC CTG MA.17. J. Natl. Cancer Inst..

[187] Jakesz R., Greil R., Gnant M., Schmid M., Kwasny W., Kubista E., Mlineritsch B., Tausch C., Stierer M., Hofbauer F. (2007). Extended adjuvant therapy with anastrozole among postmenopausal breast cancer patients: Results from the randomized Austrian Breast and Colorectal Cancer Study Group Trial 6a. J. Natl. Cancer Inst..

[188] Mamounas E.P., Bandos H., Lembersky B.C., Jeong J.H., Geyer C.E., Rastogi P., Fehrenbacher L., Graham M.L., Chia S.K., Brufsky A.M. (2019). Use of letrozole after aromatase inhibitor-based therapy in postmenopausal breast cancer (NRG Oncology/NSABP B-42): A randomised, double-blind, placebo-controlled, phase 3 trial. Lancet Oncol..

[189] Jackson H.W., Fischer J.R., Zanotelli V.R., Ali H.R., Mechera R., Soysal S.D., Moch H., Muenst S., Varga Z., Weber W.P. (2020). The single-cell pathology landscape of breast cancer. Nature.

[190] Rozenblatt-Rosen O., Regev A., Oberdoerffer P., Nawy T., Hupalowska A., Rood J.E., Ashenberg O., Cerami E., Coffey R.J., Demir E. (2020). The human tumor atlas network: Charting tumor transitions across space and time at single-cell resolution. Cell.

[191] Wu S.Z., Al-Eryani G., Roden D.L., Junankar S., Harvey K., Andersson A., Thennavan A., Wang C., Torpy J.R., Bartonicek N. (2021). A single-cell and spatially resolved atlas of human breast cancers. Nat. Genet..

[192] Lei Y., Tang R., Xu J., Wang W., Zhang B., Liu J., Yu X., Shi S. (2021). Applications of single-cell sequencing in cancer research: Progress and perspectives. J. Hematol. Oncol..

[193] Fathi M., Martinez-Paniagua M., Rezvan A., Montalvo M.J., Mohanty V., Chen K., Mani S.A., Varadarajan N. (2023). Identifying signatures of EV secretion in metastatic breast cancer through functional single-cell profiling. iScience.

[194] Cani A.K., Dolce E.M., Darga E.P., Hu K., Liu C.J., Rae J.M., Thomas D.G., Tomlins S.A., Chinnaiyan A.M., Udager A.M. (2022). Serial monitoring of single-cell circulating tumor cell genomics in metastatic lobular breast cancer to identify precision and immuno-oncology biomarkers with therapeutic implications. Cancer Res..

[195] Bartoschek M., Oskolkov N., Bocci M., Lövrot J., Larsson C., Sommarin M., Madsen C.D., Lindgren D., Pekar G., Karlsson G. (2018). Spatially and functionally distinct subclasses of breast cancer-associated fibroblasts revealed by single cell RNA sequencing. Nat. Commun..

[196] Grosselin K., Durand A., Marsolier J., Poitou A., Marangoni E., Nemati F., Dahmani A., Lameiras S., Reyal F., Frenoy O. (2019). High-throughput single-cell ChIP-seq identifies heterogeneity of chromatin states in breast cancer. Nat. Genet..

[197] Venkatachalapathy S., Jokhun D.S., Andhari M., Shivashankar G.V. (2021). Single cell imaging-based chromatin biomarkers for tumor progression. Sci. Rep..

[198] Zhang X., Marjani S.L., Hu Z., Weissman S.M., Pan X., Wu S. (2016). Single-Cell Sequencing for Precise Cancer Research: Progress and ProspectsSingle-Cell Sequencing of Cancer. Cancer Res..

[199] Wagner J., Rapsomaniki M.A., Chevrier S., Anzeneder T., Langwieder C., Dykgers A., Rees M., Ramaswamy A., Muenst S., Soysal S.D. (2019). A single-cell atlas of the tumor and immune ecosystem of human breast cancer. Cell.

[200] Ding S., Chen X., Shen K. (2020). Single-cell RNA sequencing in breast cancer: Understanding tumor heterogeneity and paving roads to individualized therapy. Cancer Commun..

[201] Zhang S., Gong C., Ruiz-Martinez A., Wang H., Davis-Marcisak E., Deshpande A., Popel A.S., Fertig E.J. (2021). Integrating single cell sequencing with a spatial quantitative systems pharmacology model spQSP for personalized prediction of triple-negative breast cancer immunotherapy response. Immunoinformatics.

[202] Yu J., Guo Z., Wang L. (2023). Progress and Challenges of Immunotherapy Predictive Biomarkers for Triple Negative Breast Cancer in the Era of Single-Cell Multi-Omics. Life.

[203] Sant G.R., Knopf K.B., Albala D.M. (2017). Live-single-cell phenotypic cancer biomarkers-future role in precision oncology?. NPJ Precis. Oncol..

[204] Xing K., Zhang B., Wang Z., Zhang Y., Chai T., Geng J., Qin X., Chen X.S., Zhang X., Xu C. (2023). Systemically Identifying Triple-Negative Breast Cancer Subtype-Specific Prognosis Signatures, Based on Single-Cell RNA-Seq Data. Cells.

[205] Anatskaya O.V., Vinogradov A.E. (2022). Polyploidy as a Fundamental Phenomenon in Evolution, Development, Adaptation and Diseases. Int. J. Mol. Sci..

[206] Anatskaya O.V., Vinogradov A.E. (2022). Polyploidy and Myc Proto-Oncogenes Promote Stress Adaptation via Epigenetic Plasticity and Gene Regulatory Network Rewiring. Int. J. Mol. Sci..

[207] Erenpreisa J., Salmina K., Anatskaya O., Cragg M.S. (2022). Paradoxes of cancer: Survival at the brink. Semin. Cancer Biol..

[208] Du M., Zhang S., Liu X., Xu C., Zhang X. (2022). Nondiploid cancer cells: Stress, tolerance and therapeutic inspirations. Biochim. Biophys. Acta Rev. Cancer.

[209] Casotti M.C., Meira D.D., Zetum A.S.S., Araújo B.C., Silva D.R.C.D., Santos E.V.W., Garcia F.M., Paula F., Santana G.M., Louro L.S. (2023). Computational Biology Helps Understand How Polyploid Giant Cancer Cells Drive Tumor Success. Genes.

[210] Anatskaya O.V., Vinogradov A.E. (2021). Whole-Genome Duplications in Evolution, Ontogeny, and Pathology: Complexity and Emergency Reserves. Mol. Biol..

[211] Erenpreisa J., Salmina K., Anatskaya O., Vinogradov A., Cragg M.S. (2022). The Enigma of cancer resistance to treatment. Org. J. Biol. Sci..

[212] Zhou X., Zhou M., Zheng M., Tian S., Yang X., Ning Y., Li Y., Zhang S. (2022). Polyploid giant cancer cells and cancer progression. Front. Cell Dev. Biol..

[213] Herbein G. (2022). Tumors and Cytomegalovirus: An Intimate Interplay. Viruses.

[214] Song Y., Zhao Y., Deng Z., Zhao R., Huang Q. (2021). Stress-Induced Polyploid Giant Cancer Cells: Unique Way of Formation and Non-Negligible Characteristics. Front. Oncol..

[215] Zhang H., Ma H., Yang X., Fan L., Tian S., Niu R., Yan M., Zheng M., Zhang S. (2022). Cell Fusion-Related Proteins and Signaling Pathways, and Their Roles in the Development and Progression of Cancer. Front. Cell Dev. Biol..

[216] El Baba R., Pasquereau S., Haidar Ahmad S., Diab-Assaf M., Herbein G. (2022). Oncogenic and Stemness Signatures of the High-Risk HCMV Strains in Breast Cancer Progression. Cancers.

[217] Nehme Z., Pasquereau S., Haidar Ahmad S., El Baba R., Herbein G. (2022). Polyploid giant cancer cells, EZH2 and Myc upregulation in mammary epithelial cells infected with high-risk human cytomegalovirus. EBioMedicine.

[218] Liu K., Zheng M., Zhao Q., Zhang K., Li Z., Fu F., Zhang H., Du J., Li Y., Zhang S. (2020). Different p53 genotypes regulating different phosphorylation sites and subcellular location of CDC25C associated with the formation of polyploid giant cancer cells. J. Exp. Clin. Cancer Res..

[219] Liu K., Lu R., Zhao Q., Du J., Li Y., Zheng M., Zhang S. (2019). Association and clinicopathologic significance of p38MAPK-ERK-JNK-CDC25C with polyploid giant cancer cell formation. Med. Oncol..

[220] Tagal V., Roth M.G. (2021). Loss of Aurora Kinase Signaling Allows Lung Cancer Cells to Adopt Endoreplication and Form Polyploid Giant Cancer Cells That Resist Antimitotic Drugs. Cancer Res..

[221] Zhang J., Qiao Q., Xu H., Zhou R., Liu X. (2022). Human cell polyploidization: The good and the evil. Semin. Cancer Biol..

[222] Bowers R.R., Andrade M.F., Jones C.M., White-Gilbertson S., Voelkel-Johnson C., Delaney J.R. (2022). Autophagy modulating therapeutics inhibit ovarian cancer colony generation by polyploid giant cancer cells (PGCCs). BMC Cancer.

[223] Niu N., Yao J., Bast R.C., Sood A.K., Liu J. (2021). IL-6 promotes drug resistance through formation of polyploid giant cancer cells and stromal fibroblast reprogramming. Oncogenesis.

[224] Silva E.G., Lawson B.C., Ramalingam P., Liu J., Shehabeldin A., Marques-Piubelli M.L., Malpica A. (2022). Precursors in the ovarian stroma: Another pathway to explain the origin of ovarian serous neoplasms. Hum. Pathol..

[225] Yart L., Bastida-Ruiz D., Allard M., Dietrich P.Y., Petignat P., Cohen M. (2022). Linking unfolded protein response to ovarian cancer cell fusion. BMC Cancer.

[226] Thura M., Ye Z., Al-Aidaroos A.Q., Xiong Q., Ong J.Y., Gupta A., Li J., Guo K., Ang K.H., Zeng Q. (2021). PRL3 induces polypoid giant cancer cells eliminated by PRL3-zumab to reduce tumor relapse. Commun. Biol..

[227] Fu F., Chen L., Yang X., Fan L., Zhang M., Chen S., Zheng M., Gao M., Zhang S. (2022). PLK4 is a key molecule in the formation of PGCCs and promotes invasion and migration of progeny cells derived from PGCCs. J. Cancer.

[228] Li Z., Zheng M., Zhang H., Yang X., Fan L., Fu F., Fu J., Niu R., Yan M., Zhang S. (2021). Arsenic Trioxide Promotes Tumor Progression by Inducing the Formation of PGCCs and Embryonic Hemoglobin in Colon Cancer Cells. Front. Oncol..

[229] Peerapen P., Sueksakit K., Boonmark W., Yoodee S., Thongboonkerd V. (2022). *ARID1A* knockdown enhances carcinogenesis features and aggressiveness of Caco-2 colon cancer cells: An *in vitro* cellular mechanism study. J. Cancer.

[230] Zhao Q., Zhang K., Li Z., Zhang H., Fu F., Fu J., Zheng M., Zhang S. (2021). High Migration and Invasion Ability of PGCCs and Their Daughter Cells Associated With the Nuclear Localization of S100A10 Modified by SUMOylation. Front. Cell Dev. Biol..

[231] Liu H.T., Xia T., You Y.W., Zhang Q.C., Ni H.S., Liu Y.F., Liu Y.R., Xu Y.Q., You B., Zhang Z.X. (2021). Characteristics and clinical significance of polyploid giant cancer cells in laryngeal carcinoma. Laryngoscope Investig. Otolaryngol..

[232] You B., Xia T., Gu M., Zhang Z., Zhang Q., Shen J., Fan Y., Yao H., Pan S., Lu Y. (2022). AMPK-mTOR-Mediated Activation of Autophagy Promotes Formation of Dormant Polyploid Giant Cancer Cells. Cancer Res..

[233] Lu P., White-Gilbertson S., Beeson G., Beeson C., Ogretmen B., Norris J., Voelkel-Johnson C. (2021). Ceramide Synthase 6 Maximizes p53 Function to Prevent Progeny Formation from Polyploid Giant Cancer Cells. Cancers.

[234] White-Gilbertson S., Lu P., Esobi I., Echesabal-Chen J., Mulholland P.J., Gooz M., Ogretmen B., Stamatikos A., Voelkel-Johnson C. (2022). Polyploid giant cancer cells are dependent on cholesterol for progeny formation through amitotic division. Sci. Rep..

[235] Pustovalova M., Blokhina T., Alhaddad L., Chigasova A., Chuprov-Netochin R., Veviorskiy A., Filkov G., Osipov A.N., Leonov S. (2022). CD44+ and CD133+ Non-Small Cell Lung Cancer Cells Exhibit DNA Damage Response Pathways and Dormant Polyploid Giant Cancer Cell Enrichment Relating to Their p53 Status. Int. J. Mol. Sci..

[236] Voelkel-Johnson C. (2022). Sphingolipids in embryonic development, cell cycle regulation, and stemness—Implications for polyploidy in tumors. Semin. Cancer Biol..

